# Supplementary material for: I/O-efficient iterative matrix inversion with photonic integrated circuits
Source: Nat Commun. 2024 Jul 15;15:5926. doi: 10.1038/s41467-024-50302-3 (PMC11251023; doi:10.1038/s41467-024-50302-3)
Supplement: Supplementary file 1 — Supplementary Information [file 41467_2024_50302_MOESM1_ESM.pdf]

Supplemental Information

I/O-efficient iterative matrix inversion with photonic integrated circuits

Minjia Chen[1], Yizhi Wang[1], Chunhui Yao[1], Adrian Wonfor[1], Shuai Yang[1], Richard Penty[1], Qixiang Cheng[1,2*]

[1] Centre for Photonic Systems, Electrical Engineering Division, Department of Engineering, University of Cambridge, Cambridge, CB3 0FA, UK

[2] GlitterinTech Limited, Xuzhou, China

*E-mail: qc223@cam.ac.uk

1. The Richardson method

1.1 Choice of the Richardson method

Matrix inversion methods can be mainly classified into direct and iterative methods[1,2]. Direct inversion methods are mainly decomposition-based, which include Gaussian elimination, lower-upper decomposition, Cholesky decomposition, QR decomposition and singular value decomposition, etc. Conventionally matrix inversions are computed in digital electronic computers using direct methods. However, direct methods require a large amount of memory access, which is not suitable for photonic processors. In contrast, iterative methods compute the inverse by first choosing an initial guess and then updating it in each iteration until convergence. Iterative methods include classical methods such as Jacobi, Gauss-Seidel, Successive overrelaxation, and Richardson methods, and gradient descent methods such as steepest descent, and conjugate gradient methods. For classical iterative methods, outputs from the previous iteration can be directly sent back to inputs for next-iteration's computation, which enables iterative photonic processors to reach an ultra-fast processing speed if the loopback paths are short enough. Among all the classical iterative methods, Richardson method has the simplest form and the least constraints on the matrix to be inverted, justifying the rationality of choosing the Richardson method for our photonic iterative processor[3].

1.2 Convergence of the Richardson method

The convergence of an iterative algorithm is reached when the error between the ideal solution and the computation results after certain iterations is below an acceptable value. For the Richardson method, the error matrix after k iterations, $\mathbf{E}^{(k)}$, can be written as:

$$\mathbf{E}^{(k)} = \mathbf{X}^{(k)} - \mathbf{X}^* = (\mathbf{I}_N - \omega\mathbf{A}) \cdot \left(\mathbf{X}^{(k-1)} - \mathbf{X}^*\right) = \cdots = (\mathbf{I}_N - \omega\mathbf{A})^{(k+1)} \cdot \mathbf{X}^* \tag{S1}$$

where $\mathbf{X}^{(k+1)}$ is the computation results after $(k+1)$ iterations, $\mathbf{X}^*$ is the ideal solution which satisfies $\mathbf{X}^* = (\mathbf{I}_N - \omega\mathbf{A})\mathbf{X}^* + \mathbf{X}^{(0)}$, and $\mathbf{X}^{(0)} = \omega\mathbf{I}_N$ is the initial input. The norm of the error matrix is:

$$\left\|\mathbf{E}^{(k)}\right\| = \left\|(\mathbf{I}_N - \omega\mathbf{A})^{(k+1)} \cdot \mathbf{X}^*\right\| \le \|\mathbf{I}_N - \omega\mathbf{A}\|^{(k+1)} \cdot \|\mathbf{X}^*\| \tag{S2}$$

The convergence of the Richardson method is defined as:

$$\left\|\mathbf{E}^{(k)}\right\| / \|\mathbf{X}^*\| \le \|\mathbf{I}_N - \omega\mathbf{A}\|^{(k+1)} \le \varepsilon \tag{S3}$$

where ε is a preset acceptable error level.

Eq. (S3) is satisfied when $\|\mathbf{I}_N - \omega\mathbf{A}\| < 1$. For Richardson method, we previously propose a "Min-Max" algorithm to compute the optimal ω that generates the least convergence iterations[3]. However, for most practical applications including ridge regression and wireless communications, $\mathbf{A}$ is generally assumed to be positive definite, with N eigenvalues, $0 < \lambda_1 \le \lambda_2 \le \cdots \le \lambda_N$. $\|\mathbf{I}_N - \omega\mathbf{A}\| < 1$ is satisfied when $\left|1 - \omega\lambda_j\right| < 1\ (j = 1,2,\ldots,N)$, which means ω needs to satisfy $0 < \omega < \omega_{\max} = 2/\lambda_N$. The optimal ω for fastest convergence (minimizing $\left|1 - \omega\lambda_j\right|$) is $\omega_{\text{opt}} = 2/(\lambda_1 + \lambda_N)$ [1,2]. Considering $\left\|\mathbf{I}_N - \omega_{\text{opt}}\mathbf{A}\right\| = \max_j\left|1 - \omega_{\text{opt}}\lambda_j\right| = |(\lambda_N - \lambda_1)/(\lambda_N + \lambda_1)|$, and redefining the iteration number $P = k + 1$ respectively, the number of

iterations for convergence under an error level ε is:

$$P = \left\lceil \frac{\ln(1/\varepsilon)}{\ln[|(\lambda_N+\lambda_1)/(\lambda_N-\lambda_1)|]} \right\rceil \tag{S4}$$

where $\lceil\cdot\rceil$ is the ceiling operation.

1.3 A comparison of C-to-IO ratios

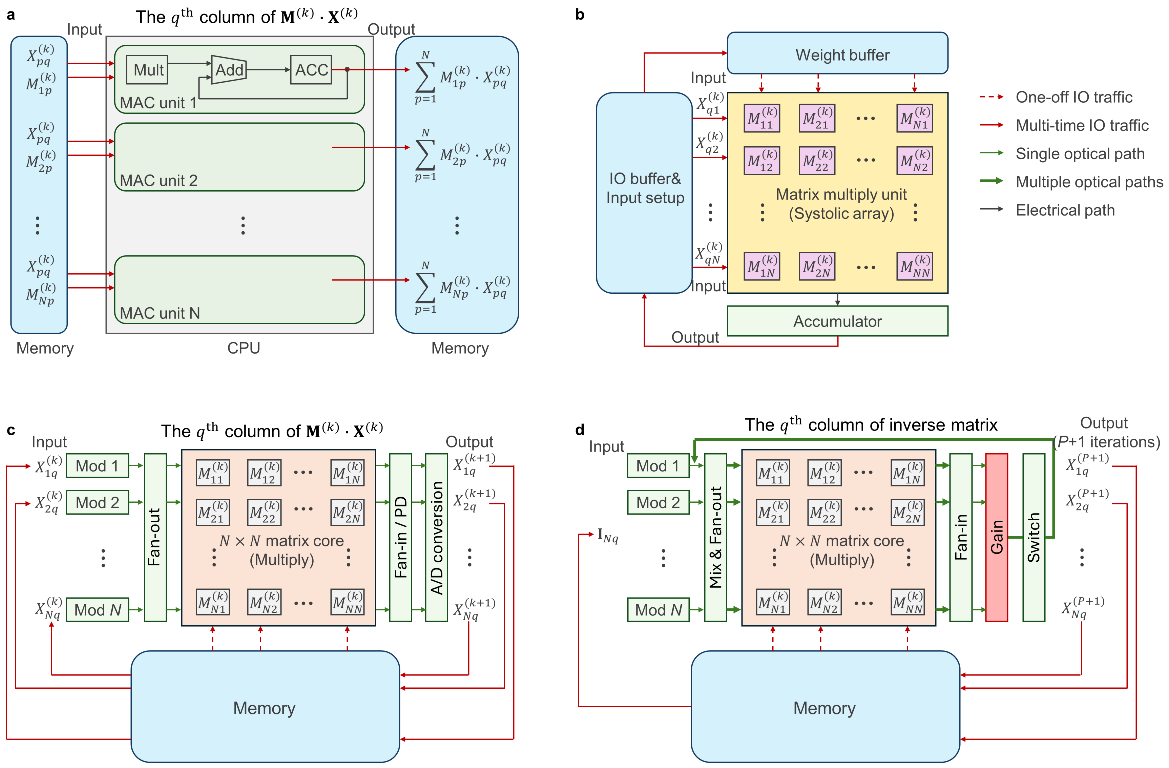

Figure S1 | Illustration of data flows for inverting an $N \times N$ matrix in (a) CPU (b) TPU (c) PSP and (d) PIP. For each set of input and output data, the CPU and PSP compute one column of the matrix-matrix multiplication result in one iteration of the Richardson method. The TPU generates the matrix-matrix multiplication result in a systolic way[6]. The PIP directly computes one column of the inverse matrix. $\mathbf{M}^{(k)}$, and $\mathbf{X}^{(k)}$ represent the weight matrix, $\mathbf{M}$, and input matrix, $\mathbf{X}$, at the kth iteration. $M_{ij}^{(k)}$, and $X_{ij}^{(k)}$ represent the element in the ith row and j^{th} column of the weight matrix, $\mathbf{M}$, and input matrix, $\mathbf{X}$, at the kth iteration. $\mathbf{I}_{Nq}$ is the qth column of the $N \times N$ identity matrix.

The computation-to-IO (C-to-IO) ratios of digital computers, photonic single-pass processors (PSPs) and photonic iterative processors (PIPs) are compared to illustrate the advantage of the PIP architecture in alleviating the IO bottleneck and speeding up matrix inversion tasks. The C-to-IO ratios are characterized by evaluating the task of inverting an $N \times N$ non-singular matrix using the iterative Richardson method: $\mathbf{X}^{(k+1)} = (\mathbf{I}_N - \omega\mathbf{A})\mathbf{X}^{(k)} + \omega\mathbf{I}_N$ $(k = 0,1,2,\ldots)$, where $\mathbf{M} = \mathbf{I}_N - \omega\mathbf{A}$ is the weight matrix, $\mathbf{X}^{(0)} = \omega\mathbf{I}_N$ is the initial input, and $\mathbf{X}^{(k)}$ is the input data in the kth iteration. In each iteration, the method requires a matrix-matrix multiplication and a matrix-matrix addition. Since here a matrix-matrix addition only involves N operations and addition is less time-consuming than multiplication in digital computers, we only consider multiply-and-accumulate (MAC) as the elementary operation. Assume the iteration is converged after $(P+1)$ iterations, which is determined by the matrix property and the required accuracy (See Supplementary 1.2 for the derived formula). We choose CPU[4] (central processing unit, representing the traditional general-purpose serial computing scheme), TPU[5,6] (tensor processing unit, representing the state of art matrix multiplication unit that computes parallelly and employs a systolic architecture to reuse the input data), PSP[7], and our proposed PIP as 4 platforms to compare the C-to-IO ratios and the results are listed in Table 1 in the main text. The total IO traffic should also include the memory access for loading the weight matrix. The corresponding data flow is illustrated in Fig. S1. As shown in Table 1, the PIP has

the highest C-to-IO ratio among the 4 computing platforms and is thus an I/O efficient approach for accelerating matrix inversions. A theoretical proof is provided below:

$$\text{C-to-IO (PIP)} - \text{C-to-IO (CPU)} = \frac{N^3P}{2N^2+N} - \frac{N}{3} = \frac{N^2(P-1)}{3(2N^2+N)} \geq 0, \text{ for } P \geq 1.$$

$$\text{C-to-IO (PIP)} - \text{C-to-IO (TPU/PSP)} = \frac{N^3P}{2N^2+N} - \frac{NP}{2P+1} = \frac{N^2P(2NP-N-1)}{(2N^2+N)(2P+1)} > 0, \text{ for } N \geq 2, P > 0.$$

In the following, two major application cases are chosen to illustrate the C-to-IO ratio enhancement of the PIP in practice, which are ridge regression and MIMO (multiple-input and multiple-output) precoding technology. The ridge regression task is to solve $\widehat{\boldsymbol{\beta}} = (\mathbf{X}^T\mathbf{X} + \lambda\mathbf{I})^{-1}\mathbf{X}^T\mathbf{y}$, where $\widehat{\boldsymbol{\beta}}$ is the parameter vector to be fitted (solved), $\mathbf{X}, \mathbf{y}$ are vectors containing the independent and dependent variables respectively that encode the samples, $\mathbf{I}$ is an identity matrix and λ is the ridge parameter that can be adjusted to improve the fitting results. The MIMO precoding using the minimum mean square error (MMSE) method is: $\hat{\mathbf{x}} = (\mathbf{H}^H\mathbf{H} + \lambda\mathbf{I})^{-1}\mathbf{H}^H\mathbf{y}$, where $\hat{\mathbf{x}}$ is the estimated transmitted signal, $\mathbf{H}$ is the channel matrix, $\mathbf{y}$ is the received signal, and $\lambda = \frac{N_r}{SNR}$ is ratio between the number of transmit antennas, $N_r = N$, and the signal-to-noise ratio, SNR.

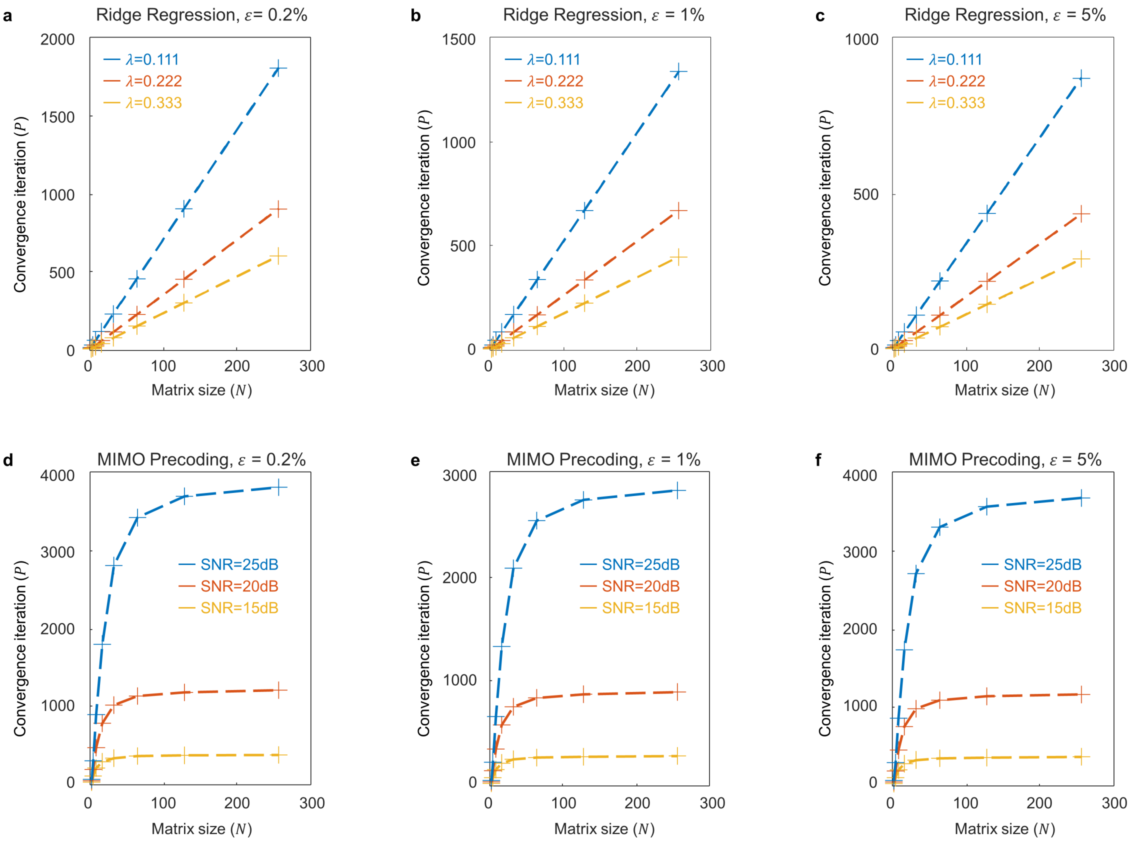

Figure S2 | Convergence iteration, P, of the PIP for different application scenarios (a) Ridge regression with an accepted error of $\varepsilon = 0.2\%$. (b) Ridge regression with an accepted error of $\varepsilon = 1\%$. (c) Ridge regression with an accepted error of $\varepsilon = 5\%$. (d) MIMO precoding with an accepted error of $\varepsilon = 0.2\%$. (e) MIMO precoding with an accepted error of $\varepsilon = 1\%$. (f) MIMO precoding with an accepted error of $\varepsilon = 5\%$. In each figure, the convergence iterations, P, for reaching the accepted error level ε are simulated and fitted for different matrix sizes and λ. λ is the ridge parameter in a ridge regression task and the ratio between number of transmit antennas and SNRs in a MIMO precoding using the minimum mean square error (MMSE) method.

The convergence iterations, P, for calculating the matrix inversions in these two applications are shown in Fig. S2 for different accepted error levels of ε = 0.2%, 1%, and 5% and different parameters, λ = 0.111, 0.222, and 0.333 which corresponds to different noise levels. $\lambda = 1$ represents an extreme case where the convergence is the fastest but the "noise level" is the same as the useful "signal level" which shall not happen in practice. For the

ridge regression task, the convergence iteration, P and matrix size, N follows a linear relationship and P is apparently much larger than N. For the MIMO precoding task, P is larger than N for almost all error levels and SNRs shown in the figure. These results indicate that our PIP has a much higher (at least N times higher for an $N \times N$ inversion) C-to-IO ratio than PSP and digital computers in these two applications. A mathematical proof is provided below.

C-to-IO (PIP) / C-to-IO (CPU) $= \frac{N^3 P}{2N^2+N} \div \frac{N}{3} = \frac{3NP}{2N+1} \geq N$, for $P \geq N$.

C-to-IO (PIP) / C-to-IO (TPU/PSP) $= \frac{N^3 P}{2N^2+N} \div \frac{NP}{2P+1} = \frac{N(2P+1)}{2N+1} \geq N$, for $P \geq N$.

2. Chip characterisation

In the following we describe methods for characterising the lossless PIP in section 2.1 – 2.3 and the coherent PIP in section 2.4 – 2.6.

2.1 Characterisation of the thermo-optic phase shifters (TOPSs)

As shown in Fig. 2a in the main text, there are 16 MZI units integrated on-chip, each encoding one element of the weight matrix. The transmission of these MZI units is thermo-optically tuned by applying voltages to heaters on each of arms. Since only real-valued computations are involved in the demonstration, only one of the two heaters of each MZI unit is needed. The characterisation set-up and characterisation result of an example MZI unit are shown in Fig. S3.

As shown in Fig. S3a, MZI units are characterised by measuring the light intensity attenuation between different combinations of input and output ports. For example, light is coupled into the chip through port 9 and coupled out through port 1 when characterising m_{23}. A 50ns light pulse from modulating the continuous-wave laser is sent to port 9. A polarization controller (PC) is used to maximize light coupled into the chip. The pulse passes the MZI unit, m_{23}, and enters the off-chip path which includes an erbium-doped fibre amplifier (EDFA), a bandpass filter (BPF) and a 1-to-2 splitter (1×2) and detected by the photodetector (PD). PDs are integrated with transimpedance amplifiers. The converted electrical signal is acquired by a 4-channel oscilloscope (OSC), which is then recorded by a computer. The MZI transmission is changed by varying the voltage applied to the thermo-optic phase shifters (TOPS), which is realized by a customized electrical control system. A Matlab program is used to control a microcontroller (MCU), which configures the output voltage of a 16-bit digital-to-analog converter (DAC). The output voltage from DAC (dynamic range 0-8V) is then amplified through a homemade 40-channel driver circuit (×4.5 amplification) and applied to the heater via bonding wires. After applying one voltage, the computer records one photo-converted voltage acquired by the OSC. The applied voltage is automatically swept from 0 V to 36 V in a step of 43.7 mV (equivalent to a 10-bit resolution).

The characterisation result of an exemplary MZI unit, m_{23}, is exhibited in Fig. S3b-c. In Fig. S1b, orange dots represent the measured points while the green curve is the fitted result. The differences between the measured results and the fitted results are shown in Fig. S3c. The transmission (T) and voltage (V) follow relationship[8]:

$$T = a\cos(bV^2 + c) + d \tag{S5}$$

where a, b are two coefficients to be fitted. The fitting results of 16 MZI units are listed in Table S1. R^2 is a measure of the goodness of a fit ranging from 0 to 1. R^2=1 means a perfect fit.

TABLE S1 Fitted Coefficients of 16 MZI Units

	m_{11}	m_{12}	m_{13}	m_{14}	m_{21}	m_{22}	m_{23}	m_{24}	m_{31}	m_{32}	m_{33}	m_{34}	m_{41}	m_{42}	m_{43}	m_{44}
a	0.51	0.49	0.49	0.48	0.49	0.49	0.49	0.49	0.49	0.61	0.60	0.49	0.49	0.49	0.49	0.49
b	0.02	0.02	0.02	0.02	0.02	0.02	0.02	0.02	0.02	0.02	0.02	0.02	0.02	0.02	0.02	0.02
c	0.42	-0.42	-0.19	-0.21	-0.12	-0.05	0.03	0.01	-0.74	0.91	0.86	-1.13	-0.01	0.02	0.09	-0.20
d	0.53	0.51	0.51	0.51	0.51	0.50	0.51	0.51	0.50	0.62	0.61	0.51	0.50	0.51	0.51	0.51
R^2	0.9999	1.0000	1.0000	1.0000	0.9999	1.0000	1.0000	0.9999	1.0000	0.9999	0.9999	0.9999	0.9999	1.0000	1.0000	1.0000

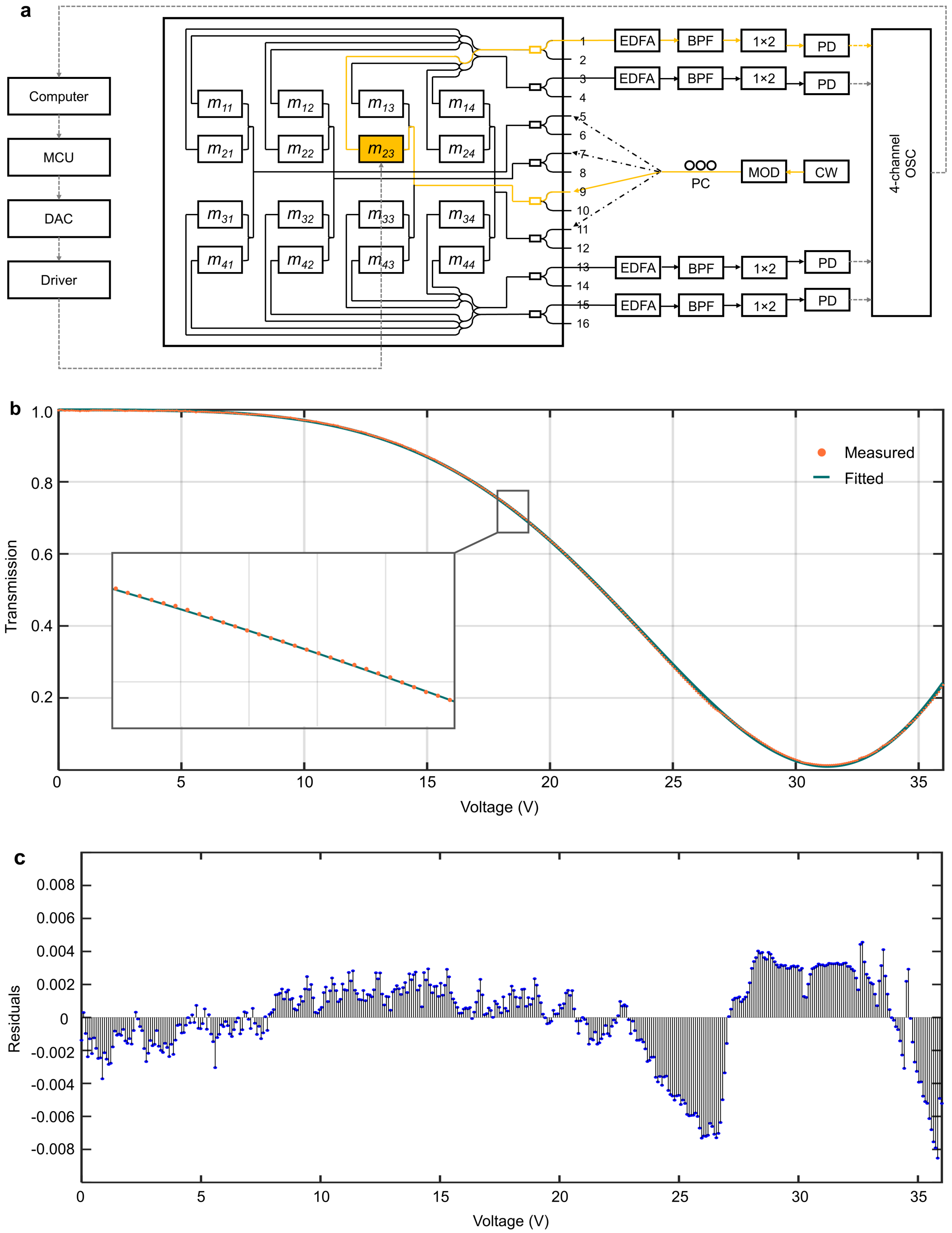

Figure S3 | Characterisation results of 16 thermo-optic phase shifters of 16 MZI units. (a) Characterisation set-up. MCU: microcontrollers. DAC: digital-to-analog converter. EDFA: Erbium-doped fibre amplifier. BPF: bandpass filter. 1×2: 1-to-2 splitter. PC: polarization controller. MOD: modulator. PD: photodetector. CW: continuous-wave laser. OSC: oscilloscope. (b) Characterisation results of an example MZI unit, m_{23}. Measured and fitted results are shown in orange dots and the green curve respectively. (c) The differences between the measured results and the fitted results shown in (b). The relatively small errors show a good agreement between the measured result and the fitted curve.

2.2 Characterisation of the relative responsivity of photodetectors

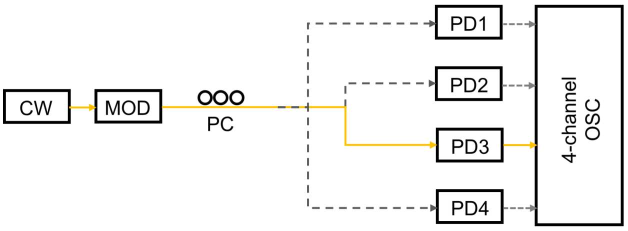

Figure S4 | Characterisation of the relative responsivity of photodetectors. Experimental set-up of the characterisation of the relative responsivities of photodetectors. A 50 ns pulse is sent to one of the four photodetectors and the photo-converted voltage is recorded in the OSC. Then fibres are disconnected from the photodetectors and connected back to repeat the previous measurement. This process is repeated 50 times for each photodetector to reduce random errors.

In order to characterise the weight bank in a close-loop form, the relative responsivities of 4 photodetectors need to be characterised as shown in Fig. S4. A 50 ns pulse is sent to one of the four photodetectors and the photo-converted voltage is recorded in the OSC. Since this setup is different from what is used in computing as shown in Fig. 2a in the main text, disconnecting and connecting fibres are required, which will normally introduce around 0.5 dB insertion loss variation. In order to improve the characterization accuracy, fibres are first disconnected from and then connected to the photodetectors before one photo-detected voltage is recorded in the OSC. This process is repeated 50 times for each photodetector to reduce random errors. If $\mathcal{R}_i$ is used to represent the responsivity of PDi (i=1, 2, 3, 4), then the relative responsivities are characterised to be $\mathcal{R}_{21} = \mathcal{R}_2/\mathcal{R}_1 = 1.12$, $\mathcal{R}_{31} = \mathcal{R}_3/\mathcal{R}_1 = 0.97$, $\mathcal{R}_{41} = \mathcal{R}_4/\mathcal{R}_1 = 0.89$.

2.3 Characterisation of the weight bank in a closed-loop form

Before computation, a matrix is loaded to the weight bank. Matrix elements need to be characterised in a closed-loop form. The actual weight is a combination of the attenuation in the weight bank, the loss in the loop including the MZI unit, and the gain in that loop. After closing the optical loops to form a recursive system and applying initial gains, the 4×4 weight bank is characterised. An optical pulse is sent to one of the MZI units and transits the optical path for a few rounds. By measuring the light intensity changes in different rounds, the attenuation coefficient of that path can be calculated. According to the corresponding element in the weight matrix that needs to be applied, the gain of that path is first adjusted. Then the required additional attenuation of the MZI unit is calculated. The applied voltage of the MZI unit is calculated according to the fitted transmission curve shown in section 2.1.

Fig. S4a-b show the setup and model for characterising m_{11}, m_{12} and m_{21}. Initially, each MZI is set to the highest transmission point. A pulse, with a power of $\mathrm{P_{in}}$ and a duration shorter than the time it takes to propagate one round in the loop, is launched into the chip. The voltage detected by PD1 at the nth output in the recursive process of characterising m_{11} is:

$$y_{1n} = \mathcal{R}_1 \mathrm{P_{in}} \frac{\alpha_1}{\alpha_2} \left(\frac{\alpha_2 m_{11} \beta_1 G_1}{2}\right)^n = \mathrm{REFV}_{11} M_{11}^n \tag{S6}$$

$\alpha_i (i = 1,2,3,4)$ and $\beta_j (j = 1,2,3,4)$ represent the coupling losses. $G_i (i = 1,2)$ represent the effective gain of the EDFA and the BPF on one path. M_{11} is obtained by exponential fitting of y_{1n} in the computer. The reference for PD1, $REFV_{11}$, when computing the first column of the inverse matrix, can then be calculated as $\mathrm{REFV}_{11} = y_{1n}/M_{11}^n$. The nth output voltages detected by PD1 and PD2 in the recursive process of characterising m_{12} and m_{21} are:

$$y_{1n} = \mathcal{R}_1 \mathrm{P_{in}} \frac{\alpha_1}{\alpha_2} \left(\frac{\alpha_2 m_{21} \beta_3 G_2}{2}\right)^n \left(\frac{\alpha_4 m_{12} \beta_1 G_1}{2}\right)^n = \mathrm{REFV}_{11} M_{21}^n M_{12}^n \tag{S7}$$

$$y_{2n} = \mathcal{R}_2 \mathrm{P_{in}} \frac{\alpha_1}{\alpha_2} \left(\frac{\alpha_2 m_{21} \beta_3 G_2}{2}\right)^n \left(\frac{\alpha_4 m_{12} \beta_1 G_1}{2}\right)^{n-1} = \mathrm{REFV}_{21} M_{21}^n M_{12}^{n-1} \tag{S8}$$

The reference for PD2, REFV_{21}, when computing the first column of the inverse matrix, is calculated according to $\mathrm{REFV}_{21} = \mathcal{R}_{21} \times \mathrm{REFV}_{11}$, M_{21} is then obtained from $M_{21} = y_{21}/\mathrm{REFV}_{21}$. $M_{21} \cdot M_{12}$ is obtained through exponential fitting of y_{1n}. M_{12} is calculated as $M_{12} = (M_{21} \cdot M_{12})/M_{21}$. The gain is adjusted by changing the EDFA current to ensure that the measured weights are larger than or equal to the corresponding matrix elements. The weights in the loop are measured again, and the required additional attenuation of the MZI unit is calculated. The computer then controls the driver to apply the required voltage according to the fitted transmission curves. This characterisation method can also be used for characterising other matrix elements. The reference voltage matrix, $[\mathrm{REFV_{ij}}, i, j = 1,2,3,4]$, is used to convert the measured output voltages into the actual elements of the computing results.

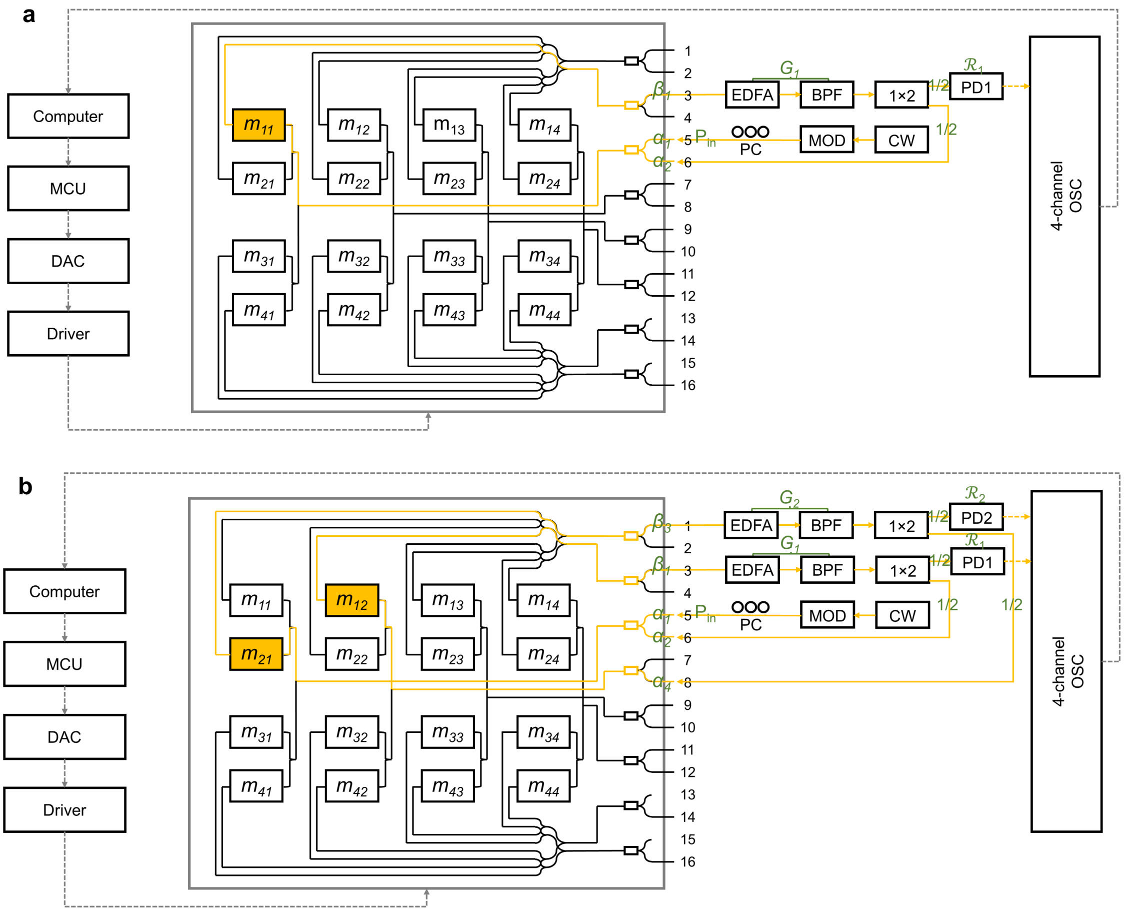

Figure S5 | Characterisation of the weight bank in a close-loop form. (a) Characterisation of m_{11} in a loop. (b) Characterisation of m_{12} and m_{21} in a loop. α_1, α_2, and α_4 represent the coupling loss when light is launched into the chip, while β_1 and β_2 represent the coupling loss when light is coupled out of the chip. $\mathrm{P_{in}}$ is the power of the input pulse. G_1 and G_2 are total signal gain introduced by the EDFA and BPF along two paths. $\mathcal{R}_1$ and $\mathcal{R}_2$ are responsivities of two photodetectors.

2.4 Characterisation of the loop switch and the input switch

The structure of the two loop switches and the input switch is shown in Fig. S6 – Structure A, which is essentially a 2×2 MZI switch with an MZI-type variable splitter[9] to ensure a high extinction ratio (ER) of the switch element. $\tau_L^2 : \kappa_L^2$, $\tau_R^2 : \kappa_R^2$, and $\tau_2^2 : \kappa_2^2$ are coupling ratios of three multimode interference (MMI) couplers, whose values are fixed after fabrication. Structure B is equivalent to structure A, with the MZI-type variable splitter replaced with a tunable coupler. For an MZI with ideal coupling ratios, the condition for reaching the "Bar" states is $\tau_1 = \tau_2$, $\phi_1 = \phi_2 + \pi$, and the condition for reaching the "Cross" state is $\tau_1 = \kappa_2$, $\phi_1 = \phi_2$. In the 2×2

high-ER MZI, θ_1 and θ_2 are swept to generate the required coupling ratio $\tau_1^2 : \kappa_1^2$.

To characterise the high-ER switch, an optical pulse is first launched into one of the input ports of the switch. The output power of one of the output ports is monitored while sweeping ϕ_1 and ϕ_2 to find the minimal transmission point. At the minimal transmission point, θ_1 and θ_2 are then swept to find the minimal and maximal transmission points which correspond to the "Cross" and "Bar" states.

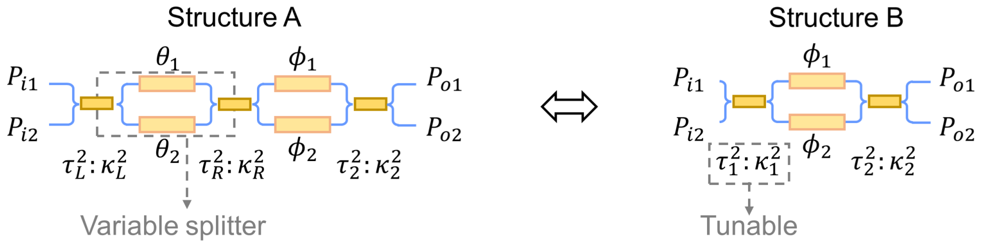

Figure S6 | Schematic of a 2×2 MZI switch with an MZI-type variable splitter. Structure A illustrates the schematic of the high-ER 2×2 MZI switch with an MZI-type variable splitter. $\tau_L^2 : \kappa_L^2$, $\tau_R^2 : \kappa_R^2$, and $\tau_2^2 : \kappa_2^2$ are coupling ratios of three multimode interference (MMI) couplers, whose values are fixed after fabrication. Structure B is equivalent to structure A, with the MZI-type variable splitter replaced with a tunable coupler.

2.5 Characterisation of the coherent detector

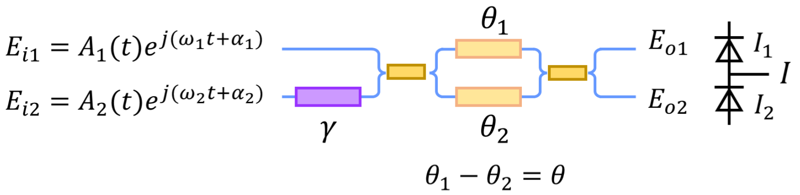

Figure S7 | Schematic and principles of the coherent detector. The MZI unit is integrated on-chip while the balanced photodetector is off-chip. By setting the phase difference between two MZI arms to $\pi/2$ and configuring the phase shifter γ to 0 and $\pi/2$, the real and imaginary parts of a complex-valued number are read out respectively.

Due to the limited space, here we integrate an MZI structure as the 90° hybrid and monitor the photo-detected current using an off-chip balanced detector as shown in Fig. S7. The working principles shown in Eq. (S9)-(S13).

$$E_{o1}(t) = je^{j(\theta/2+\theta_2+\phi)}[\sin(\theta/2)A_1(t)e^{j(\omega_1 t+\alpha_1)} + \cos(\theta/2)A_2(t)e^{j(\omega_2 t+\alpha_2+\gamma)}] \quad \text{(S9)}$$

$$E_{o2}(t) = je^{j(\theta/2+\theta_2)}[\cos(\theta/2)A_1(t)e^{j(\omega_1 t+\alpha_1)} - \sin(\theta/2)A_2(t)e^{j(\omega_2 t+\alpha_2+\gamma)}] \quad \text{(S10)}$$

$$I_1(t) = \mathcal{R}[\sin^2(\theta/2)|A_1(t)|^2 + \cos^2(\theta/2)|A_2(t)|^2 + 2\sin(\theta/2)\cos(\theta/2)A_1(t)A_2(t)\cos(\alpha_1 - \alpha_2 - \gamma)] \quad \text{(S11)}$$

$$I_2(t) = \mathcal{R}[\sin^2(\theta/2)|A_1(t)|^2 + \cos^2(\theta/2)|A_2(t)|^2 - 2\sin(\theta/2)\cos(\theta/2)A_1(t)A_2(t)\cos(\alpha_1 - \alpha_2 - \gamma)] \quad \text{(S12)}$$

$$I(t) = \mathcal{R}[-\cos\theta|A_1(t)|^2 + \cos\theta|A_2(t)|^2 + 2\sin\theta A_1(t)A_2(t)\cos(\alpha_1 - \alpha_2 - \gamma)] \quad \text{(S13)}$$

According to Eq. (S13), by setting the phase difference between two MZI arms to $\pi/2$ and configuring the phase shifter γ to 0 and $\pi/2$, the real and imaginary parts of a complex-valued number are read out respectively, which are $I(t) = 2\mathcal{R}A_1(t)A_2(t)\cos(\alpha_1 - \alpha_2)$, and $I(t) = 2\mathcal{R}A_1(t)A_2(t)\sin(\alpha_1 - \alpha_2)$.

To characterise θ, an optical pulse is launched into one input port of the coherent detector, and the photo-detected current is monitored while sweeping the voltages applied to θ_1 and leaving θ_2 untouched. The minimal and maximal current points correspond to $\theta = 0$ and $\theta = \pi/2$ respectively. Before characterising γ, θ should be set to $\pi/2$. Then two coherent pulses should be launched into two ports of the coherent detector respectively and the photo-detected current is monitored while sweeping the voltage applied to γ. The zero point corresponds to $\gamma = 0$. The maximal/minimal point corresponds to $\gamma = \pi/2$ depending on the initial phase.

2.6 Characterisation of the complex-valued weight bank in a closed-loop form

The complex-valued MZI units are characterised by launching optical pulses into two input ports respectively while calculating the ratio of output pulses after the first iteration and the initial preparation (See Supplementary 5.2). Assume the initial weight matrix is $\begin{bmatrix} m_{11} & m_{12} \\ m_{21} & m_{22} \end{bmatrix}$ and the input pulse is $\begin{bmatrix} E_i \\ 0 \end{bmatrix}$. The first and second photo-detected signals are $\begin{bmatrix} E_i \\ 0 \end{bmatrix}$ and $\begin{bmatrix} (1+m_{11})E_i \\ m_{21}E_i \end{bmatrix}$ respectively. The ratio between the output pulses after the first iteration and the initial preparation corresponds to the first column of the weight matrix. The amplitude and phase of a complex-valued number correspond to the amplitude and phase of an MZI unit, which are determined by the "differential" and "common-mode" phase shifts of two MZI arms and can be adjusted accordingly. Similarly, the second column of the weight matrix can be characterised by launching $\begin{bmatrix} 0 \\ E_i \end{bmatrix}$ via configuring the state of the input switch.

3. Numerical methods and matrix mapping

3.1 Rectangular integration technique and mapping of IE.

The rectangular integration technique is used to approximate an integral by summing a series of rectangular partitions under the curve. The integral interval $[a, b]$ is typically divided equally into N subintervals. Each rectangle has a width equal to the length of the subinterval and a height corresponding to the function's value at the midpoint of the subinterval. The Fredholm integral equation of the second kind solved in the main text, $\mathrm{f}(t) = 1 + \int_{-1}^{1} \frac{1}{5}\sqrt{t^2+s^2}\,\mathrm{f}(s)ds$, can be discretized as follows:

$$\mathrm{f}(t_i) = \mathrm{c}(t_i) + \frac{b-a}{N} \cdot \sum_{j=1}^{N} \mathrm{K}(t_i, s_j)\mathrm{f}(s_j) \tag{S14}$$

In this case, $N = 8$, $i, j = 1, 2, \ldots, 8$, $[a, b] = [-1, 1]$, $c(t) = 1$, $\mathrm{K}(t, s) = \frac{1}{5}\sqrt{t^2+s^2}$. $\mathrm{f}(t)$ is the function to be solved. Eq. (S14) can then be expressed in matrix form as $\mathbf{f} = \mathbf{c} + \frac{b-a}{N}\mathbf{Kf}$. The solution for the 8-point sampled $f(t)$ is $\mathbf{f} = (\mathbf{I} - \frac{b-a}{N}\mathbf{K})^{-1}\mathbf{c}$, where $\mathbf{f}$ is an 8×1 vector to be solved, $\mathbf{I}$ is an 8×8 unity matrix, $\frac{b-a}{N}\mathbf{K}$ is the 8×8 scaled sampled kernel function (also serving as the weight matrix to be loaded into the weight bank), and $\mathbf{c}$ is an 8×1 vector with eight elements of "1"s. In the demonstration, the values of t_i , and s_j are chosen from $[-0.875, -0.625, -0.375, -0.125, 0.125, 0.375, 0.625, 0.875]$. The scaled sampled kernel function at the 8×8 grid is as follows:

$$\mathbf{M}_{\mathbf{IE}} = \frac{b-a}{N}\mathbf{K} = \begin{bmatrix} 0.062 & 0.054 & 0.048 & 0.044 & 0.044 & 0.048 & 0.054 & 0.062 \\ 0.054 & 0.044 & 0.036 & 0.032 & 0.032 & 0.036 & 0.044 & 0.054 \\ 0.048 & 0.036 & 0.027 & 0.020 & 0.020 & 0.027 & 0.036 & 0.048 \\ 0.044 & 0.032 & 0.020 & 0.009 & 0.009 & 0.020 & 0.032 & 0.044 \\ 0.044 & 0.032 & 0.020 & 0.009 & 0.009 & 0.020 & 0.032 & 0.044 \\ 0.048 & 0.036 & 0.027 & 0.020 & 0.020 & 0.027 & 0.036 & 0.048 \\ 0.054 & 0.044 & 0.036 & 0.032 & 0.032 & 0.036 & 0.044 & 0.054 \\ 0.062 & 0.054 & 0.048 & 0.044 & 0.044 & 0.048 & 0.054 & 0.062 \end{bmatrix} \tag{S15}$$

3.2 Finite difference method and mapping of ODE and PDE.

The Finite difference (FD) method is used to solve differential equations by approximating derivatives using finite differences at evenly spaced grid points. There are three types of difference formulas: central, forward and backward differences, as shown in Eq. (S16)-(18) for the 1st order ODE.

$$\frac{dy}{dx} = \frac{y_{i+1} - y_{i-1}}{2h} \tag{S16}$$

$$\frac{dy}{dx} = \frac{y_{i+1} - y_i}{h} \tag{S17}$$

$$\frac{dy}{dx} = \frac{y_i - y_{i-1}}{h} \tag{S18}$$

where i is the index of the desired grid point, i−1 and i+1 are the indices of the neighbouring points, and h is the grid size. In this paper, we use central difference to discretize the differential equations.

The second order derivatives of ODEs can be approximated as follows:

$$\frac{d^2y}{dx^2} = \frac{y_{i+1} - 2y_i + y_{i-1}}{h^2} \tag{S19}$$

An 8-point grid is used to solve the 2nd order ODE $\frac{d^2}{dx^2}\mathrm{f}(x) - 2x\frac{d}{dx}\mathrm{f}(x) - 50\mathrm{f}(x) = -1$, with $x \in [-1,1]$ and a boundary condition of $\mathrm{f}(-1) = 1$, $\mathrm{f}(1) = 1$. The discretized equation is mapped to a matrix form: $\mathbf{A} \cdot \mathbf{f} = \mathbf{b}$:

$$\begin{bmatrix} -4.47 & 1.17 & 0 & 0 & 0 & 0 & 0 & 0 \\ 0.88 & -4.47 & 1.12 & 0 & 0 & 0 & 0 & 0 \\ 0 & 0.93 & -4.47 & 1.07 & 0 & 0 & 0 & 0 \\ 0 & 0 & 0.98 & -4.47 & 1.02 & 0 & 0 & 0 \\ 0 & 0 & 0 & 1.02 & -4.47 & 0.98 & 0 & 0 \\ 0 & 0 & 0 & 0 & 1.07 & -4.47 & 0.93 & 0 \\ 0 & 0 & 0 & 0 & 0 & 1.12 & -4.47 & 0.88 \\ 0 & 0 & 0 & 0 & 0 & 0 & 1.17 & -4.47 \end{bmatrix} \cdot \begin{bmatrix} \mathrm{f}(-0.78) \\ \mathrm{f}(-0.56) \\ \mathrm{f}(-0.33) \\ \mathrm{f}(-0.11) \\ \mathrm{f}(0.11) \\ \mathrm{f}(0.33) \\ \mathrm{f}(0.56) \\ \mathrm{f}(0.78) \end{bmatrix} = \begin{bmatrix} -0.88 \\ -0.05 \\ -0.05 \\ -0.05 \\ -0.05 \\ -0.05 \\ -0.05 \\ -0.88 \end{bmatrix} \tag{S20}$$

where $\mathbf{A}$ is the discretized coefficient matrix, $\mathbf{f}$ is a vector containing function values at discretized points, and $\mathbf{b}$ is a vector that contains the boundary conditions. Considering the facts that: 1) solving $\mathbf{A} \cdot \mathbf{f} = \mathbf{b}$ is equivalent to solving $(\omega\mathbf{A}) \cdot \mathbf{f} = \omega\mathbf{b}$, where ω is a scaling factor and 2) the weight bank can only encode values between 0 and 1, a scaling factor of $\omega = -0.22$ is chosen to appropriately encode the matrix weights. The scaled weight matrix $\mathbf{I}_N - \omega\mathbf{A}$ ($\mathbf{M_{ODE}}$) and the scaled boundary vector $\omega\mathbf{b}$ are:

$$\mathbf{M_{ODE}} = \begin{bmatrix} 0.02 & 0.26 & 0 & 0 & 0 & 0 & 0 & 0 \\ 0.19 & 0.02 & 0.25 & 0 & 0 & 0 & 0 & 0 \\ 0 & 0.20 & 0.02 & 0.24 & 0 & 0 & 0 & 0 \\ 0 & 0 & 0.21 & 0.02 & 0.23 & 0 & 0 & 0 \\ 0 & 0 & 0 & 0.23 & 0.02 & 0.21 & 0 & 0 \\ 0 & 0 & 0 & 0 & 0.24 & 0.02 & 0.20 & 0 \\ 0 & 0 & 0 & 0 & 0 & 0.25 & 0.02 & 0.19 \\ 0 & 0 & 0 & 0 & 0 & 0 & 0.26 & 0.02 \end{bmatrix} \tag{S21}$$

$$\omega\mathbf{b} = \begin{bmatrix} 0.19 \\ 0.01 \\ 0.01 \\ 0.01 \\ 0.01 \\ 0.01 \\ 0.01 \\ 0.19 \end{bmatrix} \tag{S22}$$

Solving PDEs requires discretizing the grid in 2 dimensions (2D). Again, central difference is used to approximate 2nd order partial derivatives with respect to variable x and both x, y as:

$$\frac{\partial^2 \mathrm{u}(x,y)}{\partial x^2} = \frac{\mathrm{u}_{i+1,j} - 2\mathrm{u}_{i,j} + \mathrm{u}_{i-1,j}}{h^2} \tag{S23}$$

$$\Delta\mathrm{u}(x,y) = \frac{\partial^2 \mathrm{u}(x,y)}{\partial x^2} + \frac{\partial^2 \mathrm{u}(x,y)}{\partial y^2} = \frac{\mathrm{u}_{i+1,j} + \mathrm{u}_{i,j+1} - 4\mathrm{u}_{i,j} + \mathrm{u}_{i-1,j} + \mathrm{u}_{i,j-1}}{h^2} \tag{S24}$$

where i is the index of the desired grid point, i−1 and i+1 are the indices of the neighbouring points, and h is the grid size. A 4×4 grid is used to solve the Poisson equation, $\Delta\mathbf{u}(x,y) = -2\pi^2\sin(\pi x)\sin(\pi y)$, with $x, y \in [-1,1]$ and a boundary condition of $\partial_{\mathbf{u}} = 0$ ($\mathbf{u}(-1,y) = \mathbf{u}(1,y) = \mathbf{u}(x,-1) = \mathbf{u}(x,1) = 0$). The discretized equation is then transformed into a matrix form, $\mathbf{A} \cdot \mathbf{u} = \mathbf{b}$:

$$\begin{bmatrix} -4 & 1 & 0 & 0 & 1 & 0 & 0 & 0 & 0 & 0 & 0 & 0 & 0 & 0 & 0 & 0 \\ 1 & -4 & 1 & 0 & 0 & 1 & 0 & 0 & 0 & 0 & 0 & 0 & 0 & 0 & 0 & 0 \\ 0 & 1 & -4 & 1 & 0 & 0 & 1 & 0 & 0 & 0 & 0 & 0 & 0 & 0 & 0 & 0 \\ 0 & 0 & 1 & -4 & 0 & 0 & 0 & 1 & 0 & 0 & 0 & 0 & 0 & 0 & 0 & 0 \\ 1 & 0 & 0 & 0 & -4 & 1 & 0 & 0 & 1 & 0 & 0 & 0 & 0 & 0 & 0 & 0 \\ 0 & 1 & 0 & 0 & 1 & -4 & 1 & 0 & 0 & 1 & 0 & 0 & 0 & 0 & 0 & 0 \\ 0 & 0 & 1 & 0 & 0 & 1 & -4 & 1 & 0 & 0 & 1 & 0 & 0 & 0 & 0 & 0 \\ 0 & 0 & 0 & 1 & 0 & 0 & 1 & -4 & 0 & 0 & 0 & 1 & 0 & 0 & 0 & 0 \\ 0 & 0 & 0 & 0 & 1 & 0 & 0 & 0 & -4 & 1 & 0 & 0 & 1 & 0 & 0 & 0 \\ 0 & 0 & 0 & 0 & 0 & 1 & 0 & 0 & 1 & -4 & 1 & 0 & 0 & 1 & 0 & 0 \\ 0 & 0 & 0 & 0 & 0 & 0 & 1 & 0 & 0 & 1 & -4 & 1 & 0 & 0 & 1 & 0 \\ 0 & 0 & 0 & 0 & 0 & 0 & 0 & 1 & 0 & 0 & 1 & -4 & 0 & 0 & 0 & 1 \\ 0 & 0 & 0 & 0 & 0 & 0 & 0 & 0 & 1 & 0 & 0 & 0 & -4 & 1 & 0 & 0 \\ 0 & 0 & 0 & 0 & 0 & 0 & 0 & 0 & 0 & 1 & 0 & 0 & 1 & -4 & 1 & 0 \\ 0 & 0 & 0 & 0 & 0 & 0 & 0 & 0 & 0 & 0 & 1 & 0 & 0 & 1 & -4 & 1 \\ 0 & 0 & 0 & 0 & 0 & 0 & 0 & 0 & 0 & 0 & 0 & 1 & 0 & 0 & 1 & -4 \end{bmatrix} \cdot \begin{bmatrix} u(0.2,0.2) \\ u(0.2,0.4) \\ u(0.2,0.6) \\ u(0.2,0.8) \\ u(0.4,0.2) \\ u(0.4,0.4) \\ u(0.4,0.6) \\ u(0.4,0.8) \\ u(0.6,0.2) \\ u(0.6,0.4) \\ u(0.6,0.6) \\ u(0.6,0.8) \\ u(0.8,0.2) \\ u(0.8,0.4) \\ u(0.8,0.6) \\ u(0.8,0.8) \end{bmatrix} = \begin{bmatrix} -0.27 \\ -0.44 \\ -0.44 \\ -0.27 \\ -0.44 \\ -0.71 \\ -0.71 \\ -0.44 \\ -0.44 \\ -0.71 \\ -0.71 \\ -0.44 \\ -0.27 \\ -0.44 \\ -0.44 \\ -0.27 \end{bmatrix} \tag{S25}$$

where $\mathbf{A}$ is the discretized coefficient matrix, $\mathbf{u}$ is a vector containing function values at discretized points, and $\mathbf{b}$ is a vector containing boundary conditions. To appropriately encode the matrix weights, a scaling factor $\omega = -0.25$ is chosen. The scaled weight matrix $\mathbf{I_N} - \omega\mathbf{A}$ ($\mathbf{M_{PDE}}$) and the scaled boundary vector $\omega\mathbf{b}$ are:

$$\mathbf{M_{PDE}} = \begin{bmatrix} 0 & 0.25 & 0 & 0 & 0.25 & 0 & 0 & 0 & 0 & 0 & 0 & 0 & 0 & 0 & 0 & 0 \\ 0.25 & 0 & 0.25 & 0 & 0 & 0.25 & 0 & 0 & 0 & 0 & 0 & 0 & 0 & 0 & 0 & 0 \\ 0 & 0.25 & 0 & 0.25 & 0 & 0 & 0.25 & 0 & 0 & 0 & 0 & 0 & 0 & 0 & 0 & 0 \\ 0 & 0 & 0.25 & 0 & 0 & 0 & 0 & 0.25 & 0 & 0 & 0 & 0 & 0 & 0 & 0 & 0 \\ 0.25 & 0 & 0 & 0 & 0 & 0.25 & 0 & 0 & 0.25 & 0 & 0 & 0 & 0 & 0 & 0 & 0 \\ 0 & 0.25 & 0 & 0 & 0.25 & 0 & 0.25 & 0 & 0 & 0.25 & 0 & 0 & 0 & 0 & 0 & 0 \\ 0 & 0 & 0.25 & 0 & 0 & 0.25 & 0 & 0.25 & 0 & 0 & 0.25 & 0 & 0 & 0 & 0 & 0 \\ 0 & 0 & 0 & 0.25 & 0 & 0 & 0.25 & 0 & 0 & 0 & 0 & 0.25 & 0 & 0 & 0 & 0 \\ 0 & 0 & 0 & 0 & 0.25 & 0 & 0 & 0 & 0 & 0.25 & 0 & 0 & 0.25 & 0 & 0 & 0 \\ 0 & 0 & 0 & 0 & 0 & 0.25 & 0 & 0 & 0.25 & 0 & 0.25 & 0 & 0 & 0.25 & 0 & 0 \\ 0 & 0 & 0 & 0 & 0 & 0 & 0.25 & 0 & 0 & 0.25 & 0 & 0.25 & 0 & 0 & 0.25 & 0 \\ 0 & 0 & 0 & 0 & 0 & 0 & 0 & 0.25 & 0 & 0 & 0.25 & 0 & 0 & 0 & 0 & 0.25 \\ 0 & 0 & 0 & 0 & 0 & 0 & 0 & 0 & 0.25 & 0 & 0 & 0 & 0 & 0.25 & 0 & 0 \\ 0 & 0 & 0 & 0 & 0 & 0 & 0 & 0 & 0 & 0.25 & 0 & 0 & 0.25 & 0 & 0.25 & 0 \\ 0 & 0 & 0 & 0 & 0 & 0 & 0 & 0 & 0 & 0 & 0.25 & 0 & 0 & 0.25 & 0 & 0.25 \\ 0 & 0 & 0 & 0 & 0 & 0 & 0 & 0 & 0 & 0 & 0 & 0.25 & 0 & 0 & 0.25 & 0 \end{bmatrix} \tag{S26}$$

$$\omega\mathbf{b} = \begin{bmatrix} 0.07 \\ 0.11 \\ 0.11 \\ 0.07 \\ 0.11 \\ 0.18 \\ 0.18 \\ 0.11 \\ 0.11 \\ 0.18 \\ 0.18 \\ 0.11 \\ 0.07 \\ 0.11 \\ 0.11 \\ 0.07 \end{bmatrix} \tag{S27}$$

4. Matrix inversion examples

For matrix inversion examples using the lossless PIP system, we use an alternative approach to compute the inverse matrix using a short input pulse to mitigate the phase drift in fibres. This approach is based on an equivalent expression of Richardson method as shown in Eq. (S28):

$$\mathbf{X}^{(k)} = \sum_{p=0}^{k}[(\mathbf{I}_N - \omega\mathbf{A})^{(p)} \cdot \mathbf{X}^{(0)}] \ (\mathbf{X}^{(0)} = \omega\mathbf{I}_N, k = 0,1,2,\ldots). \tag{S28}$$

An input pulse with a duration shorter than the light propagation time in a full loop can be used to encode one column of $\mathbf{X}^{(0)}$. In each iteration, the PIP effectively outputs $(\mathbf{I}_N - \omega\mathbf{A})^{(p)} \cdot \mathbf{X}^{(0)}$. The summation is then post-processed to generate final results.

4.1 Recorded waveforms for inverting $\mathbf{A}_1$.

The matrix to be inverted is $\mathbf{A}_1$ = [0.92, -0.07, -0.06, -0.06; -0.07, 0.94, -0.05, -0.04; -0.06, -0.05, 0.97, -0.02; -0.06, -0.04, -0.02, 0.99]. By choosing $\omega = 1$ to simplify preprocessing, the weight matrix to be encoded is:

$$\mathbf{I}_N - \mathbf{A}_1 = \begin{bmatrix} 0.08 & 0.07 & 0.06 & 0.06 \\ 0.07 & 0.06 & 0.05 & 0.04 \\ 0.06 & 0.05 & 0.03 & 0.02 \\ 0.06 & 0.04 & 0.02 & 0.01 \end{bmatrix} \tag{S29}$$

The experimental setup is shown in Fig. 2a in the main text. To demonstrate the computation result in each iteration, a light pulse with a duration of 50ns, which is shorter than the time for it to propagate one round in the loop (4 loop lengths are matched before the experiment), is launched into the chip. The recorded waveforms during the inversion process are shown in Fig. S8a. z_{ij} is the element in the ith row and j^{th} column of the inverse matrix. The duration of each iteration is approximately 130 ns, which is determined by the optical loop length. After 5 iterations, all 16 outputs converge, indicating that the time required to obtain one column of the inverse matrix is 650 ns. The complete inverse matrix is obtained by successively injecting four unit vectors, corresponding to a net inversion time of 2.6 μs. To calculate z_{ij}, the noise is subtracted from the pulse value. The resulting value is then divided by the reference voltage, $REFV_{ij}$, as explained in Supplementary 2.3. The final inversion results are obtained by simply adding $\mathbf{Z}^{(k)}(k = 1,2,...)$ and $\mathbf{I}_N$, which is almost computationally costless. The reference voltage matrix, **REFV**, measured inversion result, $\mathbf{A}_{meas}^{-1}$, and ideal inversion result, $\mathbf{A}_{ideal}^{-1}$ are listed below:

$$\mathbf{REFV} = \begin{bmatrix} 2.58 & 2.70 & 3.10 & 2.53 \\ 2.16 & 1.91 & 1.91 & 1.51 \\ 1.37 & 1.52 & 1.83 & 1.12 \\ 0.73 & 0.88 & 1.38 & 0.20 \end{bmatrix} \tag{S30}$$

$$\mathbf{A}_{meas}^{-1} = \begin{bmatrix} 1.09 & 0.08 & 0.07 & 0.07 \\ 0.08 & 1.07 & 0.05 & 0.05 \\ 0.07 & 0.06 & 1.04 & 0.03 \\ 0.06 & 0.05 & 0.03 & 1.05 \end{bmatrix} \tag{S31}$$

$$\mathbf{A}_{ideal}^{-1} = \begin{bmatrix} 1.10 & 0.08 & 0.07 & 0.07 \\ 0.08 & 1.07 & 0.06 & 0.05 \\ 0.07 & 0.06 & 1.04 & 0.03 \\ 0.07 & 0.05 & 0.03 & 1.02 \end{bmatrix} \tag{S32}$$

The inversion accuracy is 97.5%, calculated by: $\left(1 - \left\|\mathbf{A}_{meas}^{-1} - \mathbf{A}_{ideal}^{-1}\right\| / \left\|\mathbf{A}_{ideal}^{-1}\right\|\right) \times 100\%$.

4.2 Recorded waveforms for inverting $\mathbf{A}_2$.

The matrix to be inverted is $\mathbf{A}_2$ = [0.98, -0.26, 0, 0; -0.19, 0.98, -0.25, 0; 0, -0.2, 0.98, -0.24; 0, 0, -0.21, 0.98]. By choosing $\omega = 1$, the weight matrix to be encoded is:

$$\mathbf{I}_N - \mathbf{A}_2 = \begin{bmatrix} 0.02 & 0.26 & 0 & 0 \\ 0.19 & 0.02 & 0.25 & 0 \\ 0 & 0.20 & 0.02 & 0.24 \\ 0 & 0 & 0.21 & 0.02 \end{bmatrix} \tag{S33}$$

The recorded waveforms during the inversion process are shown in Fig. S8b. The duration of one iteration is approximately 130 ns, determined by the loop length in the experiment. After 8 iterations, all 16 outputs converge, indicating a net inversion time of 5.2 μs. The final inversion results are obtained in the same manner as described in Supplementary 4.1. The reference voltage matrix, **REFV**, measured inversion result, $\mathbf{A}_{meas}^{-1}$, and ideal inversion result, $\mathbf{A}_{ideal}^{-1}$ are listed below:

$$\boldsymbol{REFV} = \begin{bmatrix} 2.55 & 2.07 & 1.63 & 1.58 \\ 2.83 & 2.31 & 1.82 & 1.75 \\ 2.48 & 2.02 & 1.59 & 1.54 \\ 2.32 & 1.89 & 1.48 & 1.44 \end{bmatrix} \tag{S34}$$

$$\mathbf{A}_{meas}^{-1} = \begin{bmatrix} 1.08 & 0.34 & 0.07 & 0.02 \\ 0.21 & 1.16 & 0.29 & 0.08 \\ 0.04 & 0.26 & 1.15 & 0.29 \\ 0.01 & 0.05 & 0.25 & 1.09 \end{bmatrix} \tag{S35}$$

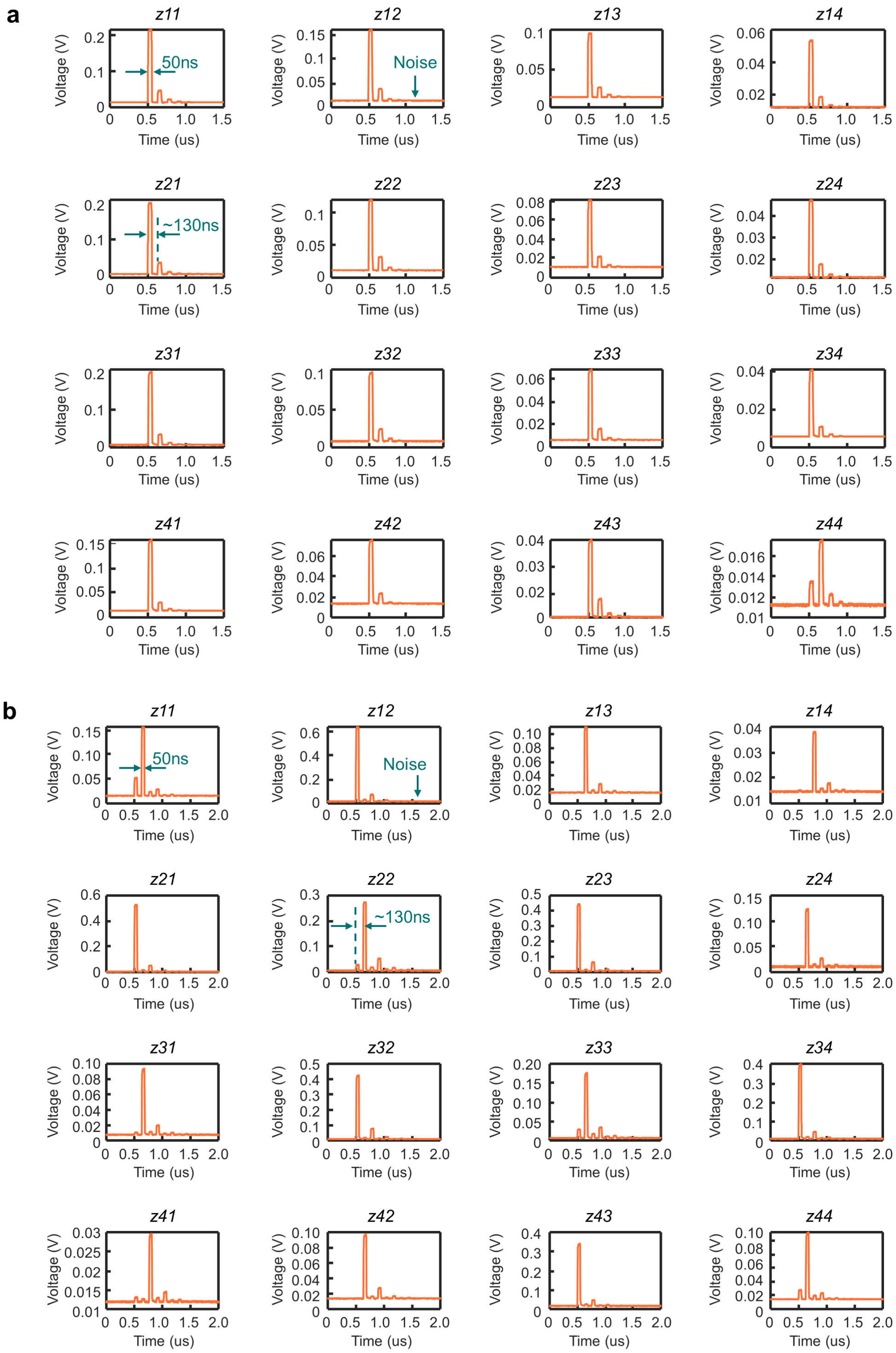

Figure S8| Recorded waveforms for matrix inversions using the lossless PIP system. A 50 ns pulse is launched into the loop to obtain the computation result in each iteration. The duration of one iteration is approximately 130 ns, which is determined by the length of the loop in the experiment. (a) Inversion process of calculating $\mathbf{A}_1^{-1}$. After 5 iterations, all 16 outputs converge, indicating an inversion time of $2.6\ \mu$s. (b) Inversion process of calculating $\mathbf{A}_2^{-1}$. After 8 iterations, all 16 outputs converge, indicating an inversion time of $5.2\ \mu$s.

$$\mathbf{A}_{ideal}^{-1} = \begin{bmatrix} 1.08 & 0.30 & 0.08 & 0.02 \\ 0.22 & 1.14 & 0.30 & 0.07 \\ 0.05 & 0.25 & 1.14 & 0.27 \\ 0.01 & 0.05 & 0.25 & 1.08 \end{bmatrix} \quad \text{(S36)}$$

The inversion accuracy is 97.0%, calculated by: $\left(1 - \|\mathbf{A}_{meas}^{-1} - \mathbf{A}_{ideal}^{-1}\| / \|\mathbf{A}_{ideal}^{-1}\|\right) \times 100\%$.

4.3 Recorded waveforms for inverting $\mathbf{A}_3$.

The matrix to be inverted is $\mathbf{A}_3 = \begin{bmatrix} 0.92 + 0.07i & 0 \\ 0 & 1.07 - 0.07i \end{bmatrix}$. By choosing $\omega = 1$ to simplify preprocessing, the weight matrix to be encoded is: $\mathbf{I}_N - \mathbf{A}_3 = \begin{bmatrix} 0.08 - 0.07i & 0 \\ 0 & -0.07 + 0.07i \end{bmatrix}$. The recorded waveforms during the inversion process are shown in Fig. S9a. z_{ij} is the element in the ith row and j^{th} column of the inverse matrix. The duration of one iteration is approximately 300 ps, determined by the integrated loop length. After 3 iterations, all 2 diagonal outputs converge, indicating a net inversion time of 1.8 ns. The final inversion results are obtained by subtracting the noise from the converged pulse value. The measured inversion result, $\mathbf{A}_{meas}^{-1}$, and ideal inversion result, $\mathbf{A}_{ideal}^{-1}$ are listed below:

$$\mathbf{A}_{meas}^{-1} = \begin{bmatrix} 1.08 - 0.08i & 0 \\ 0 & 0.93 + 0.06i \end{bmatrix} \quad \text{(S37)}$$

$$\mathbf{A}_{ideal}^{-1} = \begin{bmatrix} 1.09 - 0.07i & 0 \\ 0 & 0.93 + 0.07i \end{bmatrix} \quad \text{(S38)}$$

4.4 Recorded waveforms for inverting $\mathbf{A}_4$.

The matrix to be inverted is $\mathbf{A}_4 = \begin{bmatrix} 0.98 - 0.01i & 0.07 - 0.01i \\ 0.07 - 0.05i & 1.01 - 0.12i \end{bmatrix}$. By choosing $\omega = 1$ to simplify preprocessing, the weight matrix to be encoded is: $\mathbf{I}_N - \mathbf{A}_4 = \begin{bmatrix} 0.02 + 0.01i & -0.07 + 0.01i \\ -0.07 + 0.05i & -0.01 + 0.12i \end{bmatrix}$. The recorded waveforms during the inversion process are shown in Fig. S9b. The duration of one iteration is approximately 300 ps, determined by the integrated loop length. After 2 iterations, all 4 outputs converge, indicating a net inversion time of 1.2 ns. The final inversion results are obtained in the same manner as described in Supplementary 4.3. The measured inversion result, $\mathbf{A}_{meas}^{-1}$, and ideal inversion result, $\mathbf{A}_{ideal}^{-1}$ are listed below:

$$\mathbf{A}_{meas}^{-1} = \begin{bmatrix} 1.02 + 0.00i & -0.07 + 0.01i \\ -0.07 + 0.05i & 0.99 + 0.12i \end{bmatrix} \quad \text{(S39)}$$

$$\mathbf{A}_{ideal}^{-1} = \begin{bmatrix} 1.02 + 0.01i & -0.07 + 0.01i \\ -0.08 + 0.04i & 0.99 + 0.12i \end{bmatrix} \quad \text{(S40)}$$

4.5 Recorded waveforms for inverting $\mathbf{A}_5$.

The matrix to be inverted is $\mathbf{A}_5 = \begin{bmatrix} 0.94 + 0.03i & -0.05 - 0.06i \\ 0.02 + 0.01i & 0.98 + 0.04i \end{bmatrix}$. By choosing $\omega = 1$ to simplify preprocessing, the weight matrix to be encoded is: $\mathbf{I}_N - \mathbf{A}_5 = \begin{bmatrix} 0.06 - 0.03i & 0.05 + 0.06i \\ -0.02 - 0.01i & 0.02 - 0.04i \end{bmatrix}$. The recorded waveforms during the inversion process are shown in Fig. S9c. The duration of one iteration is approximately 300 ps, determined by the integrated loop length. After 2 iterations, all 4 outputs converge, indicating a net inversion time of 1.2 ns. The final inversion results are obtained in the same manner as described in Supplementary 4.3. The measured inversion result, $\mathbf{A}_{meas}^{-1}$, and ideal inversion result, $\mathbf{A}_{ideal}^{-1}$ are listed below:

$$\mathbf{A}_{meas}^{-1} = \begin{bmatrix} 1.06 - 0.03i & 0.05 + 0.06i \\ -0.02 - 0.01i & 1.02 - 0.04i \end{bmatrix} \quad \text{(S41)}$$

$$\mathbf{A}_{ideal}^{-1} = \begin{bmatrix} 1.06 - 0.04i & 0.05 + 0.06i \\ -0.02 - 0.01i & 1.02 - 0.04i \end{bmatrix} \quad \text{(S42)}$$

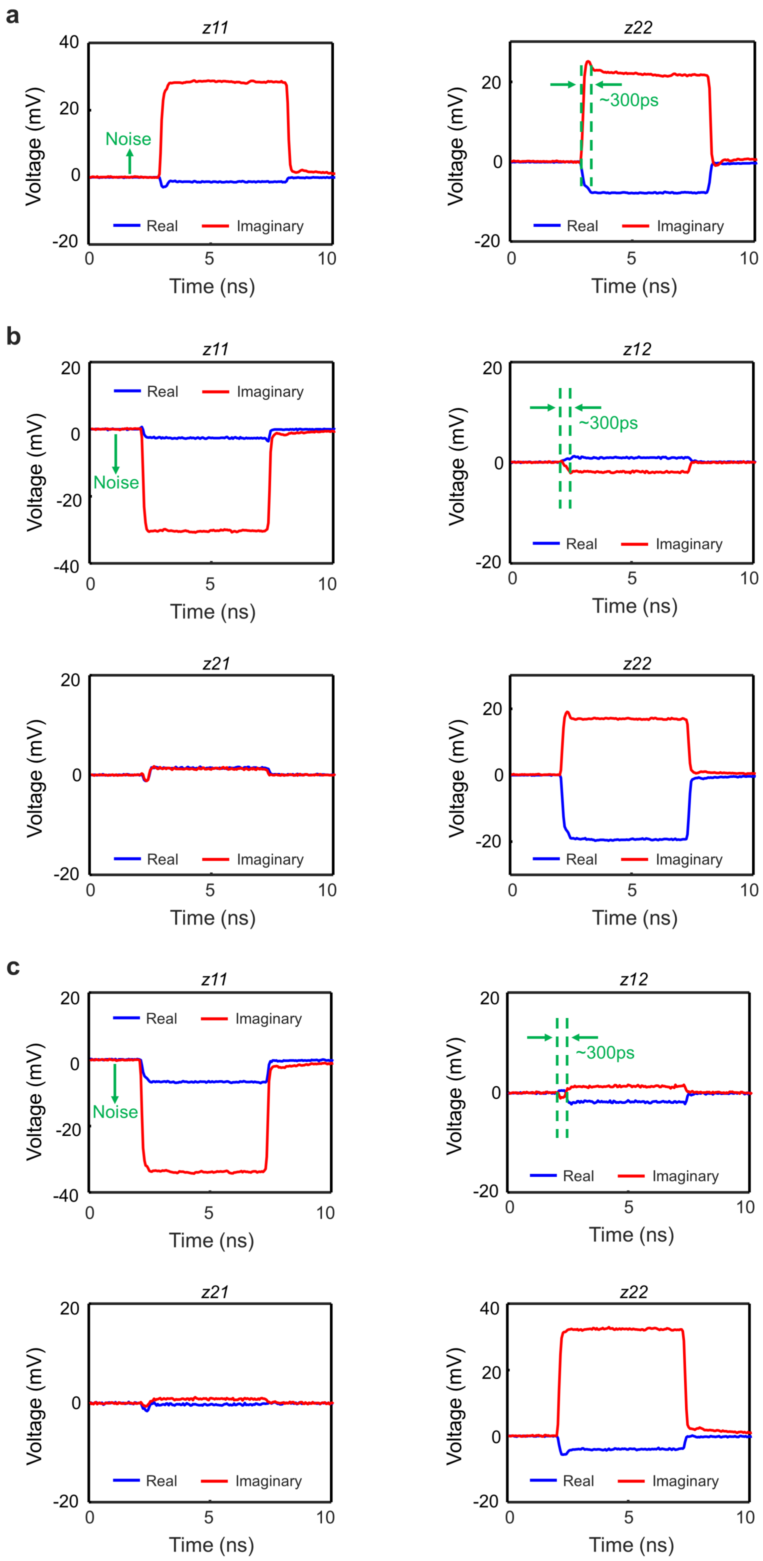

Figure S9| Recorded waveforms for matrix inversions using the coherent PIP system. A 5 ns pulse is launched into the loop to obtain the computation result in each iteration. The duration of one iteration is approximately 300 ps, which is determined by the length of the loop in the experiment. (a) Inversion process of calculating $\mathbf{A}_3^{-1}$. After 3 iterations, all 2 diagonal outputs converge, indicating an inversion time of 1.8 ns. (b) Inversion process of calculating $\mathbf{A}_4^{-1}$. After 2 iterations, all 4 outputs converge, indicating an inversion time of 1.2 ns. (c) Inversion process of calculating $\mathbf{A}_5^{-1}$. After 2 iterations, all 4 outputs converge, indicating an inversion time of 1.2 ns.

5. Equation solving examples

This paper demonstrates optically solving an integral equation (IE, Fredholm integral equation of the second kind) using an 8-point discretization, a second order ordinary differential equation (2nd order ODE) using an 8-point discretization, and a partial differential equation (PDE, Poisson equation) using a 4-point discretization. These examples involve larger-scale matrix computations performed on a 4×4 optical processor. The computations utilize block matrix computation techniques for matrix inversion, multiplication, addition and subtraction. All of these matrix computations are implemented optically. The following sections introduce the block matrix method and the solutions to three different integral and differential equations.

5.1 Matrix partition and block matrix computation principles

As the chip only integrates a 4×4 weight bank, the 8×8 or 16×16 matrix computations involved in solving integral and differential equations must be partitioned into four 4×4 blocks. If **S** and **T** are two $(2N)\times(2N)$ matrices which are both partitioned into four $N \times N$ blocks, $\mathbf{S} = \begin{bmatrix} \mathbf{A}_1 & \mathbf{B}_1 \\ \mathbf{C}_1 & \mathbf{D}_1 \end{bmatrix}$, and $\mathbf{T} = \begin{bmatrix} \mathbf{A}_2 & \mathbf{B}_2 \\ \mathbf{C}_2 & \mathbf{D}_2 \end{bmatrix}$, the block matrix computation principles are listed in the following[2]:

$$\text{Block Matrix Addition: } \mathbf{S} + \mathbf{T} = \begin{bmatrix} \mathbf{A}_1 + \mathbf{A}_2 & \mathbf{B}_1 + \mathbf{B}_2 \\ \mathbf{C}_1 + \mathbf{C}_2 & \mathbf{D}_1 + \mathbf{D}_2 \end{bmatrix} \tag{S43}$$

$$\text{Block Matrix Subtraction: } \mathbf{S} - \mathbf{T} = \begin{bmatrix} \mathbf{A}_1 - \mathbf{A}_2 & \mathbf{B}_1 - \mathbf{B}_2 \\ \mathbf{C}_1 - \mathbf{C}_2 & \mathbf{D}_1 - \mathbf{D}_2 \end{bmatrix} \tag{S44}$$

$$\text{Block Matrix Multiplication: } \mathbf{S} + \mathbf{T} = \begin{bmatrix} \mathbf{A}_1\mathbf{A}_2 + \mathbf{B}_1\mathbf{C}_2 & \mathbf{A}_1\mathbf{B}_2 + \mathbf{B}_1\mathbf{D}_2 \\ \mathbf{C}_1\mathbf{A}_2 + \mathbf{D}_1\mathbf{C}_2 & \mathbf{C}_1\mathbf{B}_2 + \mathbf{D}_1\mathbf{D}_2 \end{bmatrix} \tag{S45}$$

$$\text{Block Matrix Inversion: } \mathbf{S}^{-1} = \begin{bmatrix} \mathbf{A}_1^{-1} + \mathbf{A}_1^{-1}\mathbf{B}_1(\mathbf{D}_1 - \mathbf{C}_1\mathbf{A}_1^{-1}\mathbf{B}_1)^{-1}\mathbf{C}_1\mathbf{A}_1^{-1} & -\mathbf{A}_1^{-1}\mathbf{B}_1(\mathbf{D}_1 - \mathbf{C}_1\mathbf{A}_1^{-1}\mathbf{B}_1)^{-1} \\ -(\mathbf{D}_1 - \mathbf{C}_1\mathbf{A}_1^{-1}\mathbf{B}_1)^{-1}\mathbf{C}_1\mathbf{A}_1^{-1} & (\mathbf{D}_1 - \mathbf{C}_1\mathbf{A}_1^{-1}\mathbf{B}_1)^{-1} \end{bmatrix} \tag{S46}$$

5.2 Configurations of the PIP for different matrix computations

Fig. S10 illustrates different configurations of the PIP for different matrix computations in detail. To compute matrix addition or subtraction, i.e. $\mathbf{A} \pm \mathbf{B}$ (the subtraction process is similar to that of addition, with only a π phase shift difference), one of the matrix operands, **A**, is encoded in the weight bank before computation. In the initial preparation, a unit vector is injected to the input to retrieve one column of matrix **A**, with part of the unit vector

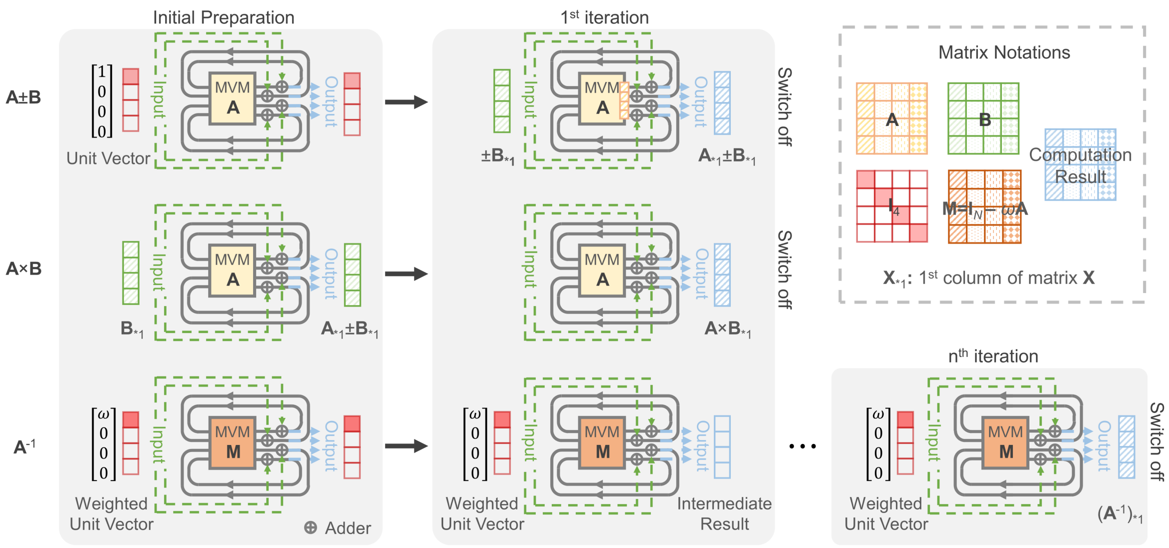

Figure S10 | Configurations of the PIP for different matrix computations. Configurations of the PIP for different matrix computations. Matrix additions, subtractions, and multiplications are solved in 1 iteration while inversions are solved in multiple iterations.

appearing at the output. The input is next updated to one column of matrix $\mathbf{B}$, which is added to the retrieved column of $\mathbf{A}$ in the 1st iteration. The loop is then terminated with the output (after splitting loss compensation) showing the addition/subtraction result of one column of $\mathbf{A}$ and $\mathbf{B}$.

To compute matrix multiplication, i.e. $\mathbf{A} \times \mathbf{B}$, $\mathbf{A}$ is again first encoded in the weight bank. Differently, in the initial preparation, one column of $\mathbf{B}$ is injected to the input, with part of it arriving directly at the output. Next, the remaining part passes the weight bank to complete a matrix-vector multiplication, and the output in the 1st iteration presents the multiplication result. The loop is afterwards terminated.

For the computation of the inverse matrix, multiple iterations are needed to let the output converge autonomously. The initial step is to load $\mathbf{M} = \mathbf{I}_N - \omega\mathbf{A}$ in the weight bank. A unit vector weighted by ω is injected to the input in the input preparation, with part of it reaching the output. In the 1st iteration, the weighted unit vector is again added to the multiplication result of $\boldsymbol{M}$ and the input vector. This procedure is repeated until the output converges at the nth iteration to one column of $\boldsymbol{A}^{-\mathbf{1}}$, and the loop is subsequently terminated.

5.3 Solutions of the IE

According to Supplementary 3.1, the solution of the 8-point sampled unknown function in the IE, $\mathbf{f}(t)$, is $\mathbf{f} = (\mathbf{I} - \frac{b-a}{N}\mathbf{K})^{-1}\mathbf{c}$, which involves an 8×8 matrix inversion and a matrix-vector multiplication. According to Eq. S46, if we partition the matrix $\mathbf{A}_{\text{IE}} = \mathbf{I} - \frac{b-a}{N}\mathbf{K}$ into $\mathbf{A}_{\text{IE}} = \begin{bmatrix} \mathbf{A}_1 & \mathbf{B}_1 \\ \mathbf{C}_1 & \mathbf{D}_1 \end{bmatrix}$, inverting $\mathbf{A}_{\text{IE}}$ is essentially inverting two 4×4 matrices, $\mathbf{A}_1$ and $\mathbf{D}_1 - \mathbf{C}_1\mathbf{A}_1^{-1}\mathbf{B}_1$, and computing a few 4×4 matrix additions, subtractions, and multiplications. The ideal solutions and measured solutions are:

$$\mathbf{f}_{\text{ideal}} = [1.61,1.49,1.39,1.32,1.32,1.39,1.49,1.61]^{\text{T}} \tag{S47}$$

$$\mathbf{f}_{\text{meas}} = [1.58,1.48,1.35,1.31,1.33,1.40,1.47,1.60]^{\text{T}} \tag{S48}$$

The computation accuracy is 98.7%, calculated by: $(1 - \|\mathbf{f}_{\text{meas}} - \mathbf{f}_{\text{ideal}}\| / \|\mathbf{f}_{\text{ideal}}\|) \times 100\%$.

5.4 Solutions of the ODE

According to Supplementary 3.2, the solution of the 8-point sampled unknown function in the ODE, $\boldsymbol{f}(x)$, is $\mathbf{f} = (\mathbf{I} - \mathbf{M}_{\text{ODE}})^{-1}(\omega\mathbf{b})$, which involves an 8×8 matrix inversion and a matrix-vector multiplication. According to Eq. S46, if we partition the matrix $\mathbf{A}_{\text{ODE}} = \mathbf{I} - \mathbf{M}_{\text{ODE}}$ into $\mathbf{A}_{\text{ODE}} = \begin{bmatrix} \mathbf{A}_1 & \mathbf{B}_1 \\ \mathbf{C}_1 & \mathbf{D}_1 \end{bmatrix}$, inverting $\mathbf{A}_{\text{ODE}}$ is essentially inverting two 4×4 matrices, $\mathbf{A}_1$ and $\mathbf{D}_1 - \mathbf{C}_1\mathbf{A}_1^{-1}\mathbf{B}_1$, and computing a few 4×4 matrix additions, subtractions, and multiplications. The intermediate computation results can be found in Additional Data. The ideal solutions and measured solutions are:

$$\mathbf{f}_{\text{ideal}} = [0.212,0.060,0.029,0.023,0.023,0.029,0.060,0.212]^{\text{T}} \tag{S49}$$

$$\mathbf{f}_{\text{meas}} = [0.213,0.057,0.028,0.023,0.023,0.030,0.063,0.214]^{\text{T}} \tag{S50}$$

The computation accuracy is 98.4%, calculated by: $(1 - \|\mathbf{f}_{\text{meas}} - \mathbf{f}_{\text{ideal}}\| / \|\mathbf{f}_{\text{ideal}}\|) \times 100\%$.

5.5 Solutions of the PDE

According to Supplementary 3.2, the solution of the 4-point sampled unknown function in the PDE, $\mathbf{u}(x, y)$, is $\mathbf{u} = (\mathbf{I} - \mathbf{M}_{\text{PDE}})^{-1}(\omega\mathbf{b})$, which involves a 16×16 matrix inversion and a matrix-vector multiplication. According to Eq. S46, if we first partition the matrix $\mathbf{A}_{\text{PDE}} = \mathbf{I} - \mathbf{M}_{\text{PDE}}$ into four 8×8 blocks as $\mathbf{A}_{\text{PDE}} = \begin{bmatrix} \mathbf{A}_1 & \mathbf{B}_1 \\ \mathbf{C}_1 & \mathbf{D}_1 \end{bmatrix}$, and then partition $\mathbf{A}_1$ into $\mathbf{A}_1 = \begin{bmatrix} \mathbf{A}_2 & \mathbf{B}_2 \\ \mathbf{C}_2 & \mathbf{D}_2 \end{bmatrix}$, partition $\mathbf{D}_1 - \mathbf{C}_1\mathbf{A}_1^{-1}\mathbf{B}_1$ into $\begin{bmatrix} \mathbf{A}_3 & \mathbf{B}_3 \\ \mathbf{C}_3 & \mathbf{D}_3 \end{bmatrix}$, inverting $\mathbf{A}_{\text{PDE}}$ is essentially inverting four 4×4 matrices, $\mathbf{A}_2$, $\mathbf{D}_2 - \mathbf{C}_2\mathbf{A}_2^{-1}\mathbf{B}_2$, $\mathbf{A}_3$, $\mathbf{D}_3 - \mathbf{C}_3\mathbf{A}_3^{-1}\mathbf{B}_3$, and computing a few 4×4 matrix additions,

subtractions, and multiplications. The ideal solutions and measured solutions are:

$$\mathbf{u}_{\text{ideal}} = \begin{bmatrix} 0.36 & 0.58 & 0.58 & 0.36 \\ 0.58 & 0.93 & 0.93 & 0.58 \\ 0.58 & 0.93 & 0.93 & 0.58 \\ 0.36 & 0.58 & 0.58 & 0.36 \end{bmatrix} \tag{S51}$$

$$\mathbf{u}_{\text{meas}} = \begin{bmatrix} 0.36 & 0.55 & 0.53 & 0.33 \\ 0.58 & 0.96 & 0.91 & 0.55 \\ 0.61 & 0.99 & 0.95 & 0.59 \\ 0.36 & 0.57 & 0.58 & 0.35 \end{bmatrix} \tag{S52}$$

The computation accuracy is 96.6%, calculated by: $(1 - \|\mathbf{u}_{\text{meas}} - \mathbf{u}_{\text{ideal}}\| / \|\mathbf{u}_{\text{ideal}}\|) \times 100\%$.

6. Demonstrated IO advantages of the PIPs

To quantify the demonstrated IO advantages of the PIP, we use saved processing time, T_{save}, saved energy consumption, E_{save}, improvement in total processing time, $\frac{\mathrm{t}_{\text{total\_PSP}}}{\mathrm{t}_{\text{total\_PIP}}}$, and improvement in C-to-IO ratio, $\frac{\text{C\_to\_IO}_{\text{PIP}}}{\text{C\_to\_IO}_{\text{PSP}}}$ as four metrics. We assume an $(2^q N) \times (2^q N)$ matrix is decomposed q times before being inverted on an $N \times N$ PIP. A total of 2^q $N \times N$ matrix inversions, $(8^q - 2^q)$ $N \times N$ matrix multiplications, and $(8^q - 2 \cdot 4^q + 2^q)$ $N \times N$ matrix additions need to be computed on the PIP. The memory access counts for $N \times N$ matrix computations on different platforms are summarised in Table S2.

TABLE S2
A COMPARISON OF MEMORY ACCESS COUNTS FOR $N \times N$ MATRIX COMPUTATIONS*

	CPU	TPU	PSP	PIP
Matrix addition	$3N^2$	$3N^2$	$3N^2$	$3N^2$
Matrix subtraction	$3N^2$	$3N^2$	$3N^2$	$3N^2$
Matrix multiplication	$3N^2$	$3N^2$	$3N^2$	$3N^2$
Matrix inversion	$3N^2 \cdot P$	$N^2 \cdot (2P+1)$	$N^2 \cdot (2P+1)$	$2N^2 + N$

* N is the matrix size. $P + 1$ is the number of iterations for the Richardson method to converge.

The memory access counts for matrix addition, subtraction, and multiplication are same for all four platforms. Thus, the inversion is the only operation that contributes to an enhancement in IO efficiency. The saved processing times, and energy consumption are expressed in Eq. (S53) – Eq. (S54).

$$\mathrm{T}_{\text{save}} = [2N^2(\textstyle\sum_{i=1}^{2^q} P_q - 1) + 2^q N(N-1)] \cdot T_0 \tag{S53}$$

$$\mathrm{E}_{\text{save}} = [2N^2(\textstyle\sum_{i=1}^{2^q} P_q - 1) + 2^q N(N-1)] \cdot E_0 \tag{S54}$$

where P_q is the convergence iterations for each decomposed matrix, T_0 and E_0 are the processing time and power consumption of a single IO access respectively.

Details about the total processing time can be found in the "Discussion" section in the main text and Supplementary 7.3. We have shown in Supplementary 1.3 that the PIP exhibits the highest C-to-IO ratio, followed by the PSP and the TPU. The CPU has the lowest C-to-IO ratio. Hence PSP is chosen as the benchmark to characterise the performance of the PIP. Considering the memory access counts listed in Table S2, the expression for the improvement in total processing time, $\frac{\mathrm{t}_{\text{total\_PSP}}}{\mathrm{t}_{\text{total\_PIP}}}$, and C-to-IO ratio, $\frac{\text{C\_to\_IO}_{\text{PIP}}}{\text{C\_to\_IO}_{\text{PSP}}}$ are expressed in Eq. (S55) – Eq. (S56).

$$\frac{\mathrm{t}_{\text{total\_PSP}}}{\mathrm{t}_{\text{total\_PIP}}} = \frac{(1.5 \cdot 8^q - 2 \cdot 4^q + 0.5 \cdot 2^q) \cdot \mathrm{t}_{\text{loop}} \cdot N + \mathrm{t}_{\text{loop}} \cdot (\sum_{i=1}^{2^q} P_q) \cdot N + (2N^2(\sum_{i=1}^{2^q} P_q) + 2^q N^2 + 6(8^q - 4^q)N^2) \cdot T_0}{(1.5 \cdot 8^q - 2 \cdot 4^q + 0.5 \cdot 2^q) \cdot \mathrm{t}_{\text{loop}} \cdot N + \mathrm{t}_{\text{loop}} \cdot (\sum_{i=1}^{2^q} P_q) \cdot N + (2^q \cdot (2N^2 + N) + 6(8^q - 4^q)N^2) \cdot T_0} \tag{S55}$$

$$\frac{\mathrm{C\_to\_IO_{PIP}}}{\mathrm{C\_to\_IO_{PSP}}} = \frac{2N^2(\sum_{i=1}^{2^q} P_q)+2^q N^2+6(8^q-4^q)N^2}{2^q\cdot\left(2N^2+N\right)+6(8^q-4^q)N^2} \quad \text{(S56)}$$

According to numerical simulations results, $\frac{t_{\mathrm{total\_PSP}}}{t_{\mathrm{total\_PIP}}}$ approaches 1 for $q > 5$, indicating that for no more than 5 decompositions, the PIP exhibits a notable IO advantage in matrix inversions tasks.

7. Performance analyses of the proposed PIP

7.1 Loop gain estimation of an $N \times N$ PIP

TABLE S3

LOOP LOSS ESTIMATION OF AN $N \times N$ PIP ON THREE PHOTONIC INTEGRATION PLATFORMS*

	SOI (dB)	SiN (dB)	IMOS (dB)
Signal splitting & combining	$20\log_{10}N+6$	$20\log_{10}N+6$	$20\log_{10}N+6$
IL of MMI	0.06[10]· $(2\log_2 N + 2)$	0.07[11]· $(2\log_2 N + 2)$	0.6[12]· $(2\log_2 N + 2)$
IL of bend	0.002[13]· $2(2\log_2 N + 2)$	0.01[11]· $2(2\log_2 N + 2)$	0.03[12] · $2(2\log_2 N + 2)$
IL of crossing	0.019[14]· $\log_2 N$	0.0156[15]· $\log_2 N$	0.2[16]· $\log_2 N$
IL of MZI	0.06·2	0.06·2	0.6·2
IL of switch	0.06·3	0.07·3	0.6·3
IL of BPF	1[17]· k_{opt}	0.22[18]· k_{opt}	0.3[12] · k_{opt}

* N is the matrix size. k_{opt} is optimal number of SOA stages that corresponds to the minimal total amplified spontaneous emission noise.

In order to encode arbitrary weight matrices, the PIP needs to operate in the lossless state, which means the required loop gain equals the loop loss. Table S3 lists the losses of different building blocks in an $N \times N$ PIP on three photonic integration platforms. IL represents insertion loss. BPF represents bandpass filter. The losses are assumed to be compensated by cascaded SOA (semiconductor optical amplifier) stages. Each SOA provides 3-15dB gain. k_{opt} is the optimal number of SOA stages that corresponds to the minimal total amplified spontaneous emission (ASE) noise. The estimated total required gain and the optimal SOA stage numbers for processor sizes ranging from 2×2 to 256×256 are shown in Fig. S11. The insertion losses of the components are the best reported values that can be found in the literature. Notably, supplying a loop gain less than the loop loss poses limitations on the encoded matrix elements. For some applications where the weight matrices do not need to be arbitrary, the required gain is less than that shown in Fig. S11.

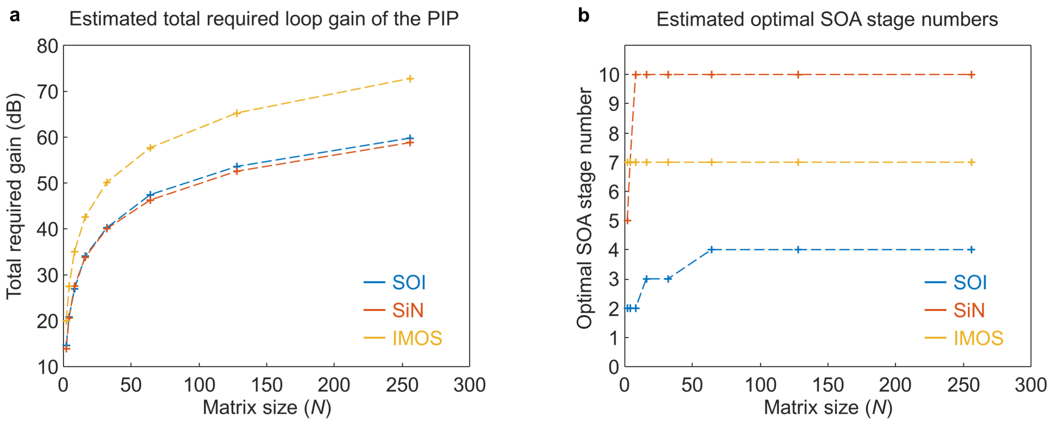

Figure S11 | Loop gain estimation of an $N \times N$ lossless PIP. (a) Estimated total required loop gain of an $N \times N$ lossless PIP. (b) Optimal SOA stage numbers for the minimal total ASE noise.

7.2 Loop length estimation of an $N \times N$ PIP

After determining the optimal number of SOA stages, the loop length of the PIP needs to be estimated to evaluate the processing time in a single iteration. Table S4 lists the lengths of each building block in the PIP based

on either reported designs in the literature or practical layout designs. The estimated total loop length, which is twice the single-pass length, is shown in Fig. S12. This is a relatively conservative estimate since the loop length can be shortened with careful design. As shown in Fig. S12, the single-pass length for a 256×256 processor remains below 100 mm. Considering the typical wafer diameters of SOI-based or SiN-based platforms to be 12-inch (300 mm) and of InP-based platforms to be 4-inch (100 mm), a 256×256 PIP can easily fit within the commercial wafer sizes, and the device footprint is not a limiting factor to its scalability.

TABLE S4

LOOP LENGTH ESTIMATION OF AN $N\times N$ PIP ON THREE PHOTONIC INTEGRATION PLATFORMS*

	**SOI (μm)**	**SiN (μm)**	**IMOS (μm)**
Adders & Splitters	11.3[19]· $(2\log_2 N + 2)$	13[11]· $(2\log_2 N + 2)$	11.8· $(2\log_2 N + 2)$
Bend	4[13]· $2(2\log_2 N + 2)$	20[11]· $2(2\log_2 N + 2)$	5· $2(2\log_2 N + 2)$
Crossing	10· $\log_2 N$	33· $\log_2 N$	16· $\log_2 N$
Phase shifter	200	200	200
MZI weight bank		$(2L_{MMI}+2L_{bend}+L_{phase\ shifter}) \cdot N$	
Switch		$3L_{MMI}+8L_{bend}+2L_{phase\ shifter}$	
SOA & BPF	(500⁺+40)· k_{opt}	(500+80)· k_{opt}	(500+48)· k_{opt}

* N is the matrix size. k_{opt} is optimal number of SOA stages that corresponds to the minimal total amplified spontaneous emission noise.

⁺ A 500 μm SOA can provide gain up to 15 dB[20].

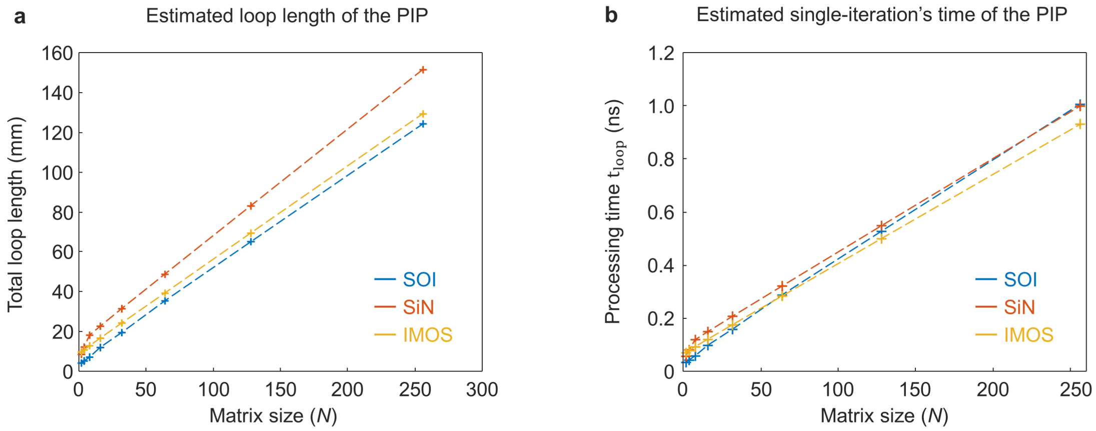

Figure S12 | Loop length and single iteration's processing time estimation of an $N \times N$ lossless PIP. (a) Estimated loop length of the PIP. The total loop length is estimated by doubling the single-pass length which is a direct summation of the length of each building block. (b) Estimated single-iteration's time of the PIP.

7.3 Processing time (Latency) estimation of an $N \times N$ PIP

The processing time of the proposed PIP consists of two parts: (1) Core processing time and (2) IO access time. The two parts are discussed below.

1) Core processing time

The core processing time refers to the duration starting from when a signal is launched into the processor until the computation results are ready for acquisition. It is determined by the single iteration's processing time of the optical signals in the loop and the number of iterations required for the Richardson method to converge to the desired accuracy. The single iteration's processing time is calculated by $t_{loop} = L_{loop} \cdot n_{eff}/c$. L_{loop} is the estimated loop length of an $N \times N$ PIP as shown in Fig. S13. n_{eff} is the effective index of the waveguide on three platforms, which are 2.43, 1.98, and 2.16 for SOI, SiN, and IMOS platforms respectively. $c = 3 \times 10^8 m/s$ is the speed of light in vacuum. The single iteration's processing time of an $N \times N$ PIP, t_{loop}, is shown in Fig. 6c in the main text, ranging from several tens of picoseconds for a 2×2 PIP to ~1 ns for a 256×256 PIP. The number of iterations for convergence under an error level ε is: $P = \left\lceil \frac{\ln(1/\varepsilon)}{\ln[|(\lambda_N+\lambda_1)/(\lambda_N-\lambda_1)|]} \right\rceil$ as derived in Supplementary 1.2. P is application dependent and is usually larger than N. The PIP architecture shown in Fig. 1 in this paper generates

the matrix inversion results one column a time, corresponding to a core processing time of $t_{core} = t_{loop} \cdot P \cdot N$. Using wavelength multiplexing techniques[3] can reduce the core processing time to $t_{core\_wdm} = t_{loop} \cdot P$.

2) IO access time

The IO traffic of the PIP system for inverting an $N \times N$ matrix includes loading weight matrix into the MZI weight bank, launching input data to the modulators, and reading out the inversion results. As shown in Table 1 in the main text, the total memory access counts for the PIP to invert an $N \times N$ matrix is $2N^2 + N$, which includes N^2 access counts for loading the weight matrix, N access counts for launching the input data, and N^2 access counts for reading out the inversion results.

Loading the weight matrix is a one-time process that consumes time scaling with N^2. After characterisation (time for characterisation is neglected since it is a one-time process), N^2 matrix elements are loaded into the on-chip weight bank for an $N \times N$ processor by applying voltages to the phase shifters. A typical way to apply one voltage is to send a data from a desktop computer to a digital-to-analogue converter (DAC) via SPI communication protocols, with a highest data transmission rate of 60 Mbps. For each voltage applied on the heater, the voltage is encoded with 16-bits, and the SPI communication used to set the voltages has a data transmission rate of 60 Mbps, which is far more time consuming than the core processing time. In an optimised electronic-photonic co-integrated design, the transmission rate between the local buffer and the DAC can reach up to 180 Gb/s[5]. Even at this high data movement speed, the weight matrix loading time remains a heavy burden compared to the core processing time. For a 256 × 256 processor, the time for loading the weight matrix is $t_{load} = \frac{N^2 \cdot B(bit)}{\text{Data Rate } (Gbit/s)} = \frac{N^2 \cdot 16bit}{180Gbit/s}$ =5825.4 ns, which is 5000 times more than the single iteration's processing time. For the ridge regression task and the MIMO precoding task shown in Fig. S2, the weight matrix loading time is at least 2.7 times and 1.3 times more than the matrix inversion time. Though one could argue that utilizing parallel data transfer paths might alleviate the IO bottleneck, this approach is achieved at the expense of higher energy consumption and increased complexity in hardware routing and control. After the voltage is applied, it takes certain time for the phase shifters to reach its stable state. For TO phase shifters, the stabilization (heating and cooling) time is at least 5 μs[21], while for electro-optic (EO) phase shifters, the stabilization time is only around 20 ps[22], which is negligible compared to the core processing time and the data movement time.

The complete $N \times N$ inverse matrix is obtained by launching N input unit vectors to the modulators, which requires to access the memory N times. Similarly, the time for inputting data is calculated as $t_{input} = \frac{N \cdot B(bit)}{\text{Data Rate } (Gbit/s)}$. To read out the inversion results, which is an $N \times N$ matrix, a total of N^2 access counts is needed, corresponding to an outputting time of $t_{output} = \frac{N^2 \cdot B(bit)}{\text{Data Rate } (Gbit/s)}$. The total IO access time of our PIP is thus estimated to be $t_{IO\_PIP} = \frac{(2N^2+N) \cdot B(bit)}{\text{Data Rate } (Gbit/s)}$. Table S5 lists the worst-case core processing time (t_{loop}) among three integration platforms, minimal/maximal iteration numbers shown in Fig. S2 (P_{Ridge}(min/max), P_{MIMO}(min/max)), minimal/maximal inversion time (t_{core_Ridge}(min/max), t_{core_MIMO}(min/max)) and IO access time (t_{IO_PIP}) of an $N \times N$ PIP.

TABLE S5
PROCESSING TIME DECOMPOSITION OF AN *N*×*N* PIP FOR MATRIX INVERSION

	t_{loop} (ns)	P_{Ridge}(min/max)	P_{MIMO}(min/max)	t_{core_Ridge}(min/max) (ns)	t_{core_MIMO}(min/max) (ns)	t_{IO_PIP} (ns)
2×2	0.07	5/17	18/72	0.7/2.4	2.5/10.1	0.89
4×4	0.08	7/34	54/310	2.3/11.0	17.4/100	3.2
8×8	0.12	12/64	104/899	11.5/61.2	99.4/859.2	12.09
16×16	0.15	21/120	144/1800	50.2/286.8	344.2/4302.3	46.93
32×32	0.21	39/233	167/2809	258.8/1546.4	1108.3/1864.3	184.89

64×64	0.32	75/457	178/3419	1543.6/9405.9	3663.6/70369	733.87
128×128	0.55	147/906	183/3690	10314/63567	12840/258899	2924.1
256×256	1.01	292/1804	187/3808	75186/464510	48150/980510	11673.6

TABLE S6
PROCESSING TIME COMPARISON BETWEEN AN N×N PIP AND AN N×N PSP FOR MATRIX INVERSION

	$t_{IO\_PSP\_Ridge}$ (min/max) (μs)	$t_{IO\_PSP\_MIMO}$ (min/max) (μs)	$t_{tot\_PIP\_Ridge}$ (min/max) (μs)	$t_{tot\_PIP\_MIMO}$ (min/max) (μs)	$t_{tot\_PSP\_Ridge}$ (min/max) (μs)	$t_{tot\_PSP\_MIMO}$ (min/max) (μs)
2×2	0.004/0.01	0.01/0.05	0.002/0.003	0.003/0.011	0.005/0.015	0.016/0.062
4×4	0.02/0.10	0.16/0.88	0.006/0.014	0.021/0.10	0.024/0.11	0.17/0.98
8×8	0.14/0.73	1.2/10.2	0.024/0.073	0.11/0.87	0.15/0.80	1.3/11.1
16×16	0.98/5.5	6.6/81.9	0.097/0.33	0.39/4.3	1.0/5.8	6.9/86.2
32×32	7.2/42.5	30.5/511.5	0.44/1.7	1.3/18.8	7.4/44.1	31.6/530.1
64×64	55.0/333.1	130.0/2490	2.3/10.1	4.4/71.1	56.5/342.5	133.6/2560.4
128×128	429.6/2640.4	534.5/10749	13.2/66.5	15.8/261.8	439.9/2703.9	547.3/11008
256×256	3407.9/21024	2184.5/44372	86.9/476.2	59.8/992.2	3483.1/21488	2232.7/45353

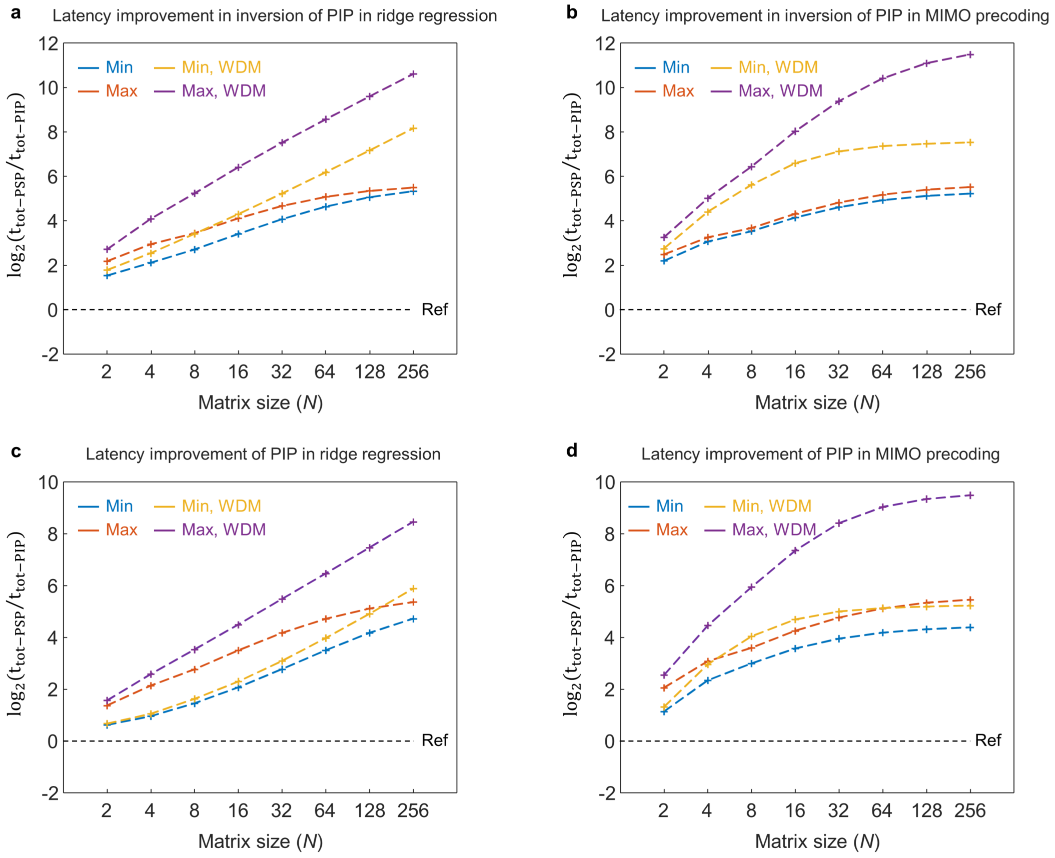

Figure S13 | Latency improvement of an $N \times N$ lossless PIP over an $N \times N$ PSP in ridge regression and MIMO precoding tasks. (a) Latency improvement in inversion operations in the ridge regression task. (b) Latency improvement in inversion operations in the MIMO precoding task. (c) Total latency improvement of the PIP in the ridge regression task. (d) Total latency improvement in the MIMO precoding task. "Min" and "Max" correspond to the minimal and maximal processing time as shown in Table S4 and S5. "WDM" represents the latency improvement when wavelength multiplexing techniques are used. Points above the "Ref" line indicates where the PIP has shorter latency than the PSP while points below the "Ref" line indicate where PIP has longer latency than the PSP. The vertical axis is the logarithm of the ratio between the total processing time of the PSP and the PIP to the base of 2.

Fig. S13a-b showcase the latency improvement of an $N \times N$ PIP over an $N \times N$ PSP (t_{tot_PSP}/t_{tot_PIP}) for implementing matrix inversions in ridge regression and MIMO precoding tasks in a more intuitive way. Fig. S13 c-d show the total latency improvement considering the time for implementing other matrix operations including one matrix-matrix addition, two matrix-matrix multiplications and one matrix-vector multiplication. Each operation can be implemented in a single pass on both the PIP and the PSP. The latency improvement for the complete task is worse than that of implementing matrix inversion solely since matrix multiplication and matrix addition have much lower C-to-IO ratio than matrix inversion. Still, up to >40 times latency improvement of the PIP over PSP is estimated for 256×256 ridge regression task and MIMO precoding task respectively thanks to the much higher C-to-IO ratio and much less reduced IO access time of the PIP. The latency improvement of the PIP when wavelength multiplexing techniques are used is also exhibited in Fig. S13, showing up to >350 and >710 times improvement for 256×256 ridge regression task and MIMO precoding task respectively. Note that we have shown in Table 1 in the main text that the state-of-the-art electronic processor has the same C-to-IO ratio as the PSP, which indicates huge potential of our proposed PIP in improving the latency of matrix-inversion-intensive tasks, especially when the problem size is large.

7.4 Inversion accuracy of an $N \times N$ PIP

As briefly mentioned in Supplementary 1.2, inversion accuracy is defined in terms of matrix norm as: $\text{accuracy} = (1-\varepsilon) \times 100\% = (1 - \|\mathbf{A}_{\text{meas}}^{-1} - \mathbf{A}_{\text{ideal}}^{-1}\| / \|\mathbf{A}_{\text{ideal}}^{-1}\|) \times 100\%$, where ε is the inversion error, $\mathbf{A}_{\text{meas}}^{-1}$ is the measured or simulated inversion results, and $\mathbf{A}_{\text{ideal}}^{-1}$ is the ideal or theoretical inversion results calculated on a 64-bit traditional digital electronic computer. Four main error sources when performing matrix inversions on the PIP include: 1) quantization error, 2) ASE noise introduced during amplification, 3) thermal and shot noise introduced during detection, and 4) phase drift in the fibres. The four error sources are analysed below, together with an interpretation of the simulated inversion accuracy results shown in Fig. 6c in the main text. Methods for mathematical modelling and choices of simulation parameters can be found in our previous paper[3].

1) Quantization error: During matrix weights loading and outputs acquisition, the digital-to-analogue and analogue-to-digital conversion (DAC and ADC) inevitably introduces quantization errors. In the lossless PIP demonstration, a 10-bit DAC and ADC resolution is used limited by the resolutions of the oscilloscope. Higher-bit resolution can be employed if the applications require higher accuracies. In the simulation results which predict the scalability of the PIP shown in Fig. 6c and Fig. S14a, a 16-bit resolution is used.

2) ASE noise introduced during amplification: ASE noise from amplification contributes most to the computation error, and it degrades the signal quality a bit in each iteration. In the lossless PIP demonstration, a bandpass filter with a 3 dB bandwidth of 0.1 nm is used to suppress the ASE noise. Narrower-bandwidth filters such as ring filters[23] can be employed if the application requires higher accuracy. Additionally, in the current partially integrated system, coupling between fibre arrays and edge couplers introduces extra loss (an average of ~12dB loss is measured per 2 facets for 4 pairs of input and output ports). Higher gain is required to compensate for the coupling loss, which in turn introduces additional ASE noise. This can be eliminated in a fully integrated chip. In the simulation shown in Fig. 6e and Fig. S13a, a filter with bandwidth of 906 kHz[24] is used. We presented the simulated inversion accuracy vs. filter bandwidth (currently reported laser power is at 19 dBm[25] but more efforts are on the way) in Fig. S14b to show the limitations in a more practical integration scheme. Figure 6e is presented again in Fig. S14a to ease the comparisons and analyses. As shown in Fig. S14, we investigate the performance of the PIP with a size up to 256×256. For PIP size not exceeding 64×64, an input signal power of > -5dBm is enough to guarantee an inversion accuracy of >90%. The input signal power, however, needs to be enhanced to maintain a certain level of SNR to handle the increased on-chip loss for the PIP of larger scales. A singular value decomposition (SVD) – based weight bank[26] can be used to improve the inversion accuracy owing to its reduced signal splitting and combining loss. Nevertheless, the computation overhead for implementing SVD is non-negligible. On the other side, a filter with 3 GHz passband is capable to guarantee a 90% inversion accuracy of a 32×32 PIP, and in order to fight against the increased ASE power, the filter passband consistently goes down

for larger-scale PIPs. For problem size exceeding 256×256, block matrix inversion techniques should be employed, considering the current PIC insertion loss and feasible laser power.

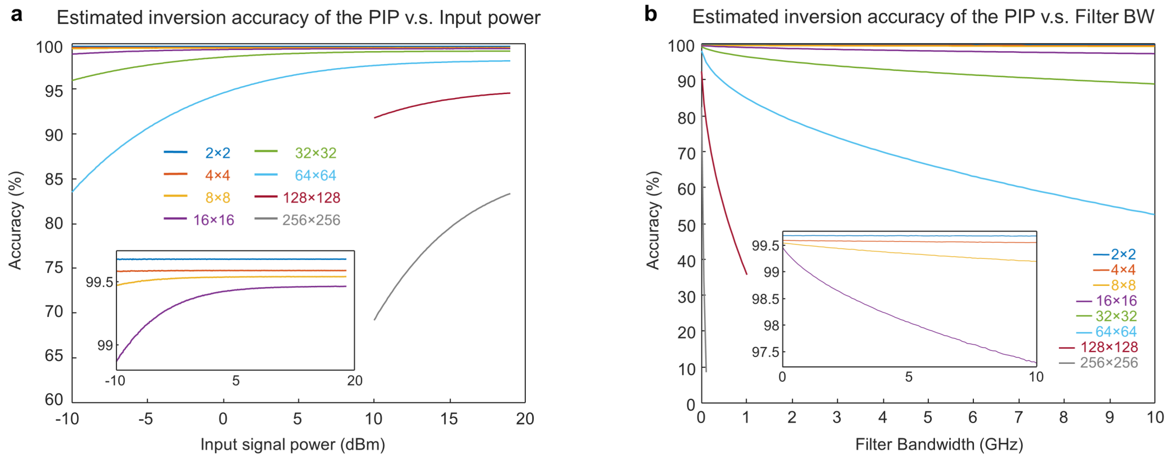

Figure S14 | Simulated inversion accuracy of an $N \times N$ lossless PIP. (a) Inversion accuracy vs. Input signal power of an $N \times N$ lossless PIP. Note the 256×256 PIP with an SVD core only consider the 6 dB splitting and adding loss. (b) Inversion accuracy vs. Filter bandwidth of an $N \times N$ lossless PIP. The matrices to be inverted are 1000 randomly generated matrices for each size.

3) Thermal and shot noise introduced during detection: thermal noise originates from the TIA circuits, while shot noise arises from photo-conversion. Thermal noise dominates when signal powers are low while shot noise dominates when signal powers are high. In the experiment, the detection noises can be subtracted from the signals in the post-processing step.

4) Phase drift in the fibres: This error source only exists in a partially integrated PIP system. The thermal-optic effect and thermally induced fibre elongation leads to intrinsic periodic fibre phase drift in common experimental conditions[27]. A typical method of dealing with the fibre phase drift is to use piezoelectrically stretched coiled fibre to compensate for the phase drift[28]. However, this method requires extra components for each phase-sensitive path and complicated control circuits and algorithms. In this paper, we use averaging to counteract the phase drift in the lossless PIP. The phase stability problem does not exist for the coherent PIP system and is not considered in the simulations.

7.5 Energy efficiency estimation of an $N \times N$ PIP

TABLE S7

ESTIMATED ENERGY CONSUMPTION OF THE PIP CORE FOR $N \times N$ MATRIX INVERSIONS*

Component	Equation
Laser	$\mathrm{E_{laser}} = \mathrm{P_{laser}}/\eta \cdot N \cdot P \cdot \mathrm{t_{loop}}$
Modulator	$E_{mod} = \mathrm{V_{mod}^2}/R \cdot N \cdot P \cdot \mathrm{t_{loop}}$
SOA	$\mathrm{E_{SOA}} = (\mathrm{P_{out}} - \mathrm{P_{in}})/\eta \cdot N^2 \cdot P \cdot \mathrm{t_{loop}}$
PD	$\mathrm{E_{PD}} = \mathcal{R}\mathrm{P_{rec}} \cdot \mathrm{V_{bias}} \cdot N^2 \cdot \mathrm{t_{loop}}$
Weight bank	$\mathrm{E_{weight}} = \mathrm{P_{heater}} \cdot N^3 \cdot P \cdot \mathrm{t_{loop}}$
Optical switch	$\mathrm{E_{switch}} = \mathrm{V_{mod}^2}/R \cdot N^2 \cdot P \cdot \mathrm{t_{loop}}$

* N is the matrix size. P is the number of iterations for the Richardson method to converge. η is the wall-plug efficiency. R is the resistance. $\mathcal{R}$ is the responsivity.

Energy efficiency of the PIP core is defined as number of operations per second per energy consumption of the PIP core. Table S7 lists the equations to estimate the energy consumption of the PIP core for inverting an $N \times N$ matrix. $\mathrm{P_{laser}}$ is the output power of the laser, which should be at least -5 dBm for PIP size not exceeding 64×64 and 10 dBm for 128×128 and 256×256 PIPs according to Supplementary 6.5. η is the wall-plug efficiency of the

laser and the semiconductor optical amplifier (SOA), which has a best reported value of around 30%[29]. V_{mod} is the driving voltage of the modulator and the optical switch, which is around 250 mV[30] for the best reported electro-optic modulators. $R = 50\Omega$ is the load impedance for modulators and optical switches. P_{out} and P_{in} are input and output powers of the SOA and are size dependent since the loop loss increases as the matrix size increases. The estimated required gain for an $N \times N$ processor is presented in Supplementary 6.1. $\mathcal{R} \approx 0.6A \cdot W^{-1}$ is the responsivity of the photodetector. P_{rec} is the received optical power at the photodetector, which should be same as P_{laser} in the lossless PIP system. The minimal required signal power P_{laser} for reaching a certain inversion accuracy is also size dependent. The inversion accuracy vs. input signal power for different-sized processor is shown in Fig. 6c. $V_{bias} \approx 3V$ is the bias voltage of photodetector. P_{heater} is the power consumption of thermo-optic phase shifters in the weight bank, whose best reported value is 0.05mW/π phase shift. t_{loop} is the core processing time in a single iteration, which depends on the loop length and the effective index of the integration platform as shown in Fig. 6a and Supplementary 6.3. Fig. S15 illustrates the estimated energy consumption and energy efficiency of the PIP core. Fig. S15a shows the energy consumption of the PIP core for computing matrix inversions in the ridge regression task and the MIMO task respectively. The minimal and maximal energy consumptions are illustrated respectively based on the minimal and maximal inversion time shown in Table S5.

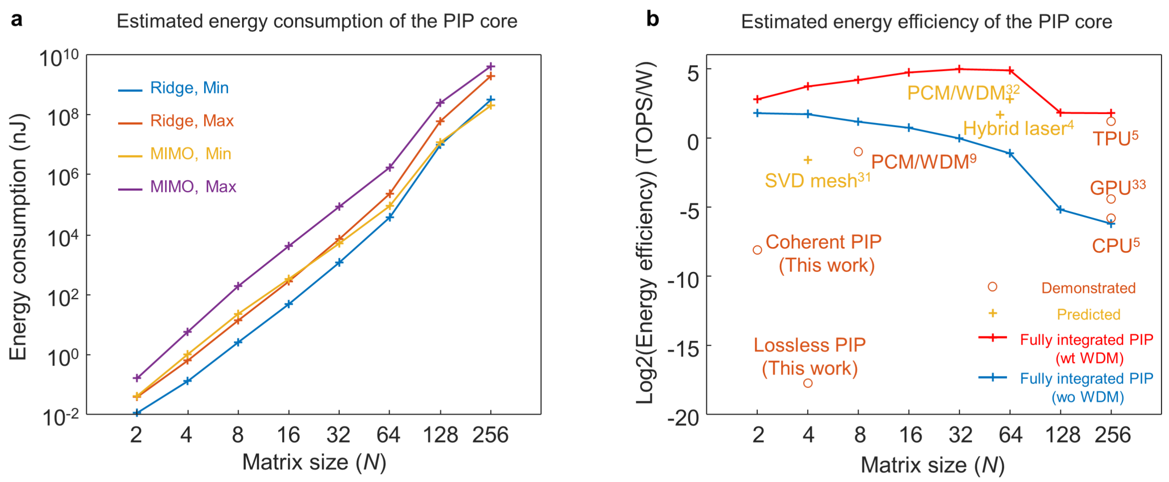

Figure S15 | Estimated energy efficiency of the lossless PIP core for inverting an $N \times N$ matrix. (a) Energy consumption of the PIP core. (b) Energy efficiency of the PIP core.

Figure. S15b shows the estimated energy efficiency of the PIP core (with and without multiplexing techniques) in terms of tera-operations per second per watt, together with the energy efficiency of the demonstrated PIP systems in this work and several state-of-the-art electronic and photonic processors to showcase the advancement in energy efficiency of our proposed PIP. Though the demonstrated coherent PIP and lossless PIP is less energy efficient than electronic processors when only the processing core is considered mainly due to the limited integration level, the predicted energy efficiency of a fully integrated lossless PIP outperforms state-of-the-art electronic and photonic processors. Considering the much-reduced IO access counts of our PIP. It is safe to say the proposed PIP is more energy efficient in matrix inversion tasks compared to traditional electronic processors and photonic processors.

8. Predicted IO advantages of the PIP for matrix-inversion-intensive applications

We evaluate the IO advantages of our PIP in matrix-inversion-intensive problems such as the MIMO precoding task and the reservoir training task. The MIMO precoding task solves $\hat{\mathbf{x}} = (\mathbf{H}^{H}\mathbf{H} + \lambda\mathbf{I})^{-1}\mathbf{H}^{H}\mathbf{y}$, where $\hat{\mathbf{x}}$ is the estimated transmitted signal, $\mathbf{H}$ is the channel matrix, $\mathbf{y}$ is the received signal, and $\lambda = \frac{N_r}{SNR}$ is ratio between the number of transmit antennas, $N_r = N$, and the signal-to-noise ratio, SNR. The reservoir training task can be reduced to a ridge regression problem[34]: $\mathbf{W}_{out} = \mathbf{Y}_{d}\mathbb{O}_{total}^{T}\left(\mathbb{O}_{total}\mathbb{O}_{total}^{T} + \lambda\mathbf{I}\right)^{-1}$, where $\mathbf{W}_{out}$ is the weight matrix,

$\mathbf{Y}_d$ is the desired output, $\mathbb{O}_{\text{total}}$ is a linear feature vector containing the training data points, λ is the ridge parameter and $\mathbf{I}$ is the identity matrix.

Matrix inversion consumes considerable computational resources in these two tasks. The matrices to be inverted in these applications are typically diagonally dominant, and their feasibility of being inverted on a PIP is verified in the experiments. To predict the advantages of using a PIP for matrix inversions in these two practical applications, we offload the matrix inversion task to a hypothetical PIP based on the previous analyses of the PIP's processing time, accuracy, and energy consumption, and implement the main model on electronic processors.

For the MIMO precoding task, the convergence iterations we use for the prediction are extracted from Fig. S2f. The problem size is chosen to be 4×4, 8×8, 128×128, and 256×256, with the first two representing the size of current MIMO systems, while the latter two representing the size of future massive MIMO systems. For the task of reservoir training 60000 MNIST samples, the convergence iterations are extracted from Fig. S2c since the simulated data used in Fig. S2a-c are processed data from the training task. The least feature number is chosen to be 10 since there are 10 digits. 60000 MNIST samples are converted to 6000 10×10 matrices by first compressing the 28×28=784 feature points into 10 feature points and then group every 10 samples into a 10×10 matrix. Typically, at least 10 epochs are required for convergence of the model which gives us a lower bound estimation of the time difference[35], corresponding to 60000 matrix inversions totally.

For MIMO precoding task, the time-varying channel matrix leads to the need for continuous matrix inversions, while for the MNIST training task, a large number of ridge parameters λ need to be chosen to find the optimal fit of the training results. The IO advantage of our PIP becomes more significant in these types of applications where a large amount of matrix inversions needs to be computed.

Reference

1. Golub, G. H. & Van Loan, C. F. *Matrix Computations*. (The Johns Hopkins University Press, Baltimore, 2013).
2. Watkins, D. S. *Fundamentals of Matrix Computations*. (Wiley-Interscience, New York, 2002).
3. Chen, M., Cheng, Q., Ayata, M., Holm, M. & Penty, R. Iterative photonic processor for fast complex-valued matrix inversion. *Photon. Res.* **10**, 2488 (2022).
4. Nahmias, M. A. *et al.* Photonic Multiply-Accumulate Operations for Neural Networks. *IEEE JOURNAL OF SELECTED TOPICS IN QUANTUM ELECTRONICS* **26**, (2020).
5. Jouppi, N. P. *et al.* In-Datacenter Performance Analysis of a Tensor Processing Unit. in *Proceedings of the 44th Annual International Symposium on Computer Architecture* 1–12 (Association for Computing Machinery, New York, NY, USA, 2017).
6. An in-depth look at Google's first Tensor Processing Unit (TPU). *Google Cloud Blog* https://cloud.google.com/blog/products/ai-machine-learning/an-in-depth-look-at-googles-first-tensor-processing-unit-tpu.
7. Zhou, H. *et al.* Photonic matrix multiplication lights up photonic accelerator and beyond. *Light Sci Appl* **11**, 30 (2022).
8. Zhang, H. *et al.* An optical neural chip for implementing complex-valued neural network. *Nat Commun* **12**, 457 (2021).
9. Suzuki, K. *et al.* Ultra-high-extinction-ratio 2 × 2 silicon optical switch with variable splitter. *Opt. Express, OE* **23**, 9086–9092 (2015).
10. Sheng, Z. *et al.* A Compact and Low-Loss MMI Coupler Fabricated With CMOS Technology. *IEEE Photonics Journal* **4**, 2272–2277 (2012).
11. Wilmart, Q. *et al.* A device library for the ultra-low loss Si3N4 platform. in *Silicon Photonics XVII* vol. 12006 80–87 (SPIE, 2022).
12. van der Tol, J. J. G. M. *et al.* InP Membrane on Silicon (IMOS) Photonics. *IEEE Journal of Quantum Electronics* **56**, 1–7 (2020).
13. Fujisawa, T., Makino, S., Sato, T. & Saitoh, K. Low-loss, compact, and fabrication-tolerant Si-wire 90° waveguide bend using clothoid and normal curves for large scale photonic integrated circuits. *Opt. Express, OE* **25**, 9150–9159 (2017).
14. Zhang, Y., Hosseini, A., Xu, X., Kwong, D. & Chen, R. T. Ultralow-loss silicon waveguide crossing using Bloch modes in index-engineered cascaded multimode-interference couplers. *Opt. Lett., OL* **38**, 3608–3611 (2013).
15. Huffman, T., Davenport, M., Belt, M., Bowers, J. E. & Blumenthal, D. J. Ultra-Low Loss Large Area Waveguide Coils for Integrated Optical Gyroscopes. *IEEE Photonics Technology Letters* **29**, 185–188 (2017).
16. Augustin, L. M., Santos, R., Latkowski, S., Mingaleev, S. & Richter, A. InP-Based Generic Foundry Platform for Photonic Integrated Circuits. *IEEE JOURNAL OF SELECTED TOPICS IN QUANTUM ELECTRONICS* **24**, (2018).
17. Morichetti, F. *et al.* Polarization-transparent silicon photonic add-drop multiplexer with wideband hitless tuneability. *Nat Commun* **12**, 4324 (2021).
18. Li, X., Gao, W., Lu, L., Chen, J. & Zhou, L. Ultra-low-loss multi-layer 8 × 8 microring optical switch. *Photon. Res., PRJ* **11**, 712–723 (2023).
19. Vázquez, C., Tapetado, A., Orcutt, J., Meng, H. C. & Ram, R. Tolerance analysis for efficient MMI devices in silicon

photonics. in *Silicon Photonics IX* vol. 8990 70–76 (SPIE, 2014).
20. Cheng, Q. Design and Experimental Characterisation of Scalable, Low-Energy Optical Switches. (University of Cambridge, 2015).
21. Liu, S. *et al.* Thermo-optic phase shifters based on silicon-on-insulator platform: state-of-the-art and a review. *Front. Optoelectron.* **15**, 9 (2022).
22. Sinatkas, G., Christopoulos, T., Tsilipakos, O. & Kriezis, E. E. Electro-optic modulation in integrated photonics. *Journal of Applied Physics* **130**, 010901 (2021).
23. Gondarenko, A., Levy, J. S. & Lipson, M. High confinement micron-scale silicon nitride high Q ring resonator. *Opt. Express* **17**, 11366 (2009).
24. Puckett, M. W. *et al.* 422 Million intrinsic quality factor planar integrated all-waveguide resonator with sub-MHz linewidth. *Nat Commun* **12**, 934 (2021).
25. Matsui, Y. *et al.* Narrow linewidth tunable semiconductor laser. in *2016 Compound Semiconductor Week (CSW) [Includes 28th International Conference on Indium Phosphide & Related Materials (IPRM) & 43rd International Symposium on Compound Semiconductors (ISCS)* 1–2 (2016).
26. Pai, S. *et al.* Experimentally realized in situ backpropagation for deep learning in photonic neural networks. *Science* **380**, 398–404 (2023).
27. Zhu, W. *et al.* The Thermal Phase Sensitivity of Both Coated and Uncoated Standard and Hollow Core Fibers Down to Cryogenic Temperatures. *Journal of Lightwave Technology* **38**, 2477–2484 (2020).
28. Jackson, D. A., Priest, R., Dandridge, A. & Tveten, A. B. Elimination of drift in a single-mode optical fiber interferometer using a piezoelectrically stretched coiled fiber. *Appl. Opt., AO* **19**, 2926–2929 (1980).
29. Sumpf, B. *et al.* High-efficient 650 nm laser bars with an output power of about 10 W and a wall-plug efficiency of 30%. in *Novel In-Plane Semiconductor Lasers V* vol. 6133 78–85 (SPIE, 2006).
30. Feng, H. *et al.* Integrated lithium niobate microwave photonic processing engine. *Nature* 1–8 (2024).
31. Shen, Y. *et al.* Deep learning with coherent nanophotonic circuits. *Nature Photon* **11**, 441–446 (2017).
32. Feldmann, J. *et al.* Parallel convolutional processing using an integrated photonic tensor core. *Nature* **589**, 52–58 (2021).
33. NVIDIA Tesla P40 Specs. *TechPowerUp* https://www.techpowerup.com/gpu-specs/tesla-p40.c2878 (2024).
34. Gauthier, D. J., Bollt, E., Griffith, A. & Barbosa, W. A. S. Next generation reservoir computing. *Nat Commun* 12, 5564 (2021).
35. Nø kland, A. Direct Feedback Alignment Provides Learning in Deep Neural Networks. in *Advances in Neural Information Processing Systems* vol. 29 (Curran Associates, Inc., 2016).
